# Intact corticostriatal control of goal-directed action in Alcohol Use Disorder: a Pavlovian-to-instrumental transfer and outcome-devaluation study

**DOI:** 10.1038/s41598-020-61892-5

**Published:** 2020-03-18

**Authors:** Tim van Timmeren, Stephanie L. Quail, Bernard W. Balleine, Dirk E. M. Geurts, Anna E. Goudriaan, Ruth J. van Holst

**Affiliations:** 1 Amsterdam UMC, Department of Psychiatry, Amsterdam Institute for Addiction Research, University of Amsterdam, Amsterdam, 1100 DD Netherlands; 2 Department of Clinical Psychology, University of Amsterdam, Amsterdam, 1018 WS The Netherlands; 3 ABC Amsterdam Brain and Cognition, University of Amsterdam, Amsterdam, 1001 NK The Netherlands; 4 Decision Neuroscience Laboratory, School of Psychology, University of New South Wales, 2052 Sydney, New South Wales Australia; 5 Donders Institute for Cognition, Brain and Behaviour, Radboud University, 6500 HB Nijmegen, Netherlands; 6 Arkin, 1033 NN Amsterdam, Netherlands

**Keywords:** Cognitive control, Motivation, Addiction

## Abstract

Deficits in instrumental, goal-directed control, combined with the influence of drug-associated Pavlovian-conditioned stimuli, are thought to influence the development and maintenance of addiction. However, direct evidence has mainly come from animal studies. We sought to establish whether alcohol use disorder (AUD) is characterized by behavioral or neurobiological deficits in (i) the integration of Pavlovian and instrumental values and (ii) goal-directed control; and (iii) whether duration or severity of AUD is associated with such deficits. The influence of cues predicting food rewards on instrumental action was assessed in a Pavlovian-to-instrumental transfer (PIT) test, measuring both specific and general PIT, and goal-directed behavior in an outcome-devaluation test. Brain activity was measured using functional MRI in 38 abstinent individuals with AUD and 22 matched healthy control individuals (HCs). We found significant specific and general PIT and outcome-devaluation effects across groups indicating goal-directed control, mediated by distinct corticostriatal signals, but no significant differences between individuals with AUD and healthy controls. Bayesian analyses provided substantial-to-strong evidence for the absence of group differences for these effects, or any relationship with duration or severity of AUD. These results suggest intact ability to integrate action-outcome associations on specific and general PIT and goal-directed learning in AUD during abstinence.

## Introduction

Alcohol use disorder (AUD) is characterized by a loss of control over alcohol consumption. Associative learning mechanisms are proposed to play a crucial role in the development and maintenance of this loss of control^1–4^. Habitual and eventually compulsive alcohol use is thought to depend largely on instrumental conditioning^5^: whereas initial drinking is thought to be goal-directed – when actions are governed by associations between actions and outcomes (e.g. drinking is pleasurable) – habitual actions are instead outcome-independent and become controlled through learned stimulus-response associations^6,7^. One way to expose which system controls responding is by outcome-devaluation. Such paradigms have been used to reveal distinct corticostriatal neural mechanisms for goal-directed actions, depending on orbitofrontal cortex (OFC), ventromedial prefrontal cortex (mPFC) and the ventral striatum, and habitual behavior depending on the premotor cortex and dorsal striatum^8–10^. Concerning AUD, habitual control over alcohol seeking indeed increases with prolonged self-administration in rodents^11^ and human AUD patients rely more on stimulus-response habits than HCs^12^.

Simultaneous with this shift in instrumental learning, repeated reinforcing effects of drug rewards become associated with the environmental stimuli that precede them through Pavlovian learning. In alcohol addiction, contextual cues (e.g., a bar) gain motivational properties that can directly influence and motivate actions (e.g., have a beer). Pavlovian-to-instrumental transfer (PIT) describes the ability of Pavlovian cues to enhance instrumental action for rewarding outcomes^13^. Failures to integrate these processes may result in reduced control of goal-directed action and facilitate drug seeking and relapse^2,6,14^. Limited evidence in addicted groups suggests that PIT can mediate how environmental stimuli promote smoking behavior in smokers^15,16^. Regarding AUD, alcohol-paired stimuli can increase alcohol-seeking in rodents^17–19^, whereas human individuals with AUD show stronger behavioral transfer effects compared to controls on a PIT task with monetary rewards and increased nucleus accumbens (NAcc) activity in relapsers compared to abstainers^20^. PIT can have two fundamentally different forms: outcome-specific transfer, demonstrated when a cue associated with a specific reward biases choice towards the same reward; and general transfer, which describes the general motivational influence of Pavlovian cues to influence instrumental actions. Lesion studies in animals have found that distinct neural structures mediate specific and general PIT, with dissociable roles for the central and basolateral amygdala^21^ and the nucleus accumbens shell and core^22^, respectively. Human neuroimaging studies have implicated the medial OFC in general transfer and different parts of the amygdala, ventral striatum and posterior putamen in both general and specific transfer^23–26^.

In addition to the shift as mentioned earlier from goal-directed to habitual control, a transition of behavioral control from specific to general transfer (produced by chronic drug exposure) has been hypothesized to characterize addiction^2^. However, whether individuals with substance use disorders indeed show reduced specific and enhanced general PIT has not been investigated. Moreover, previous human investigations into goal-directed performance in substance dependence have been limited to the use of monetary rewards and it is currently unknown whether potential deficits generalize to other types of reinforcers.

In the current study, we tested the ability of abstinent individuals with AUD ﻿ to use experienced (PIT) or predicted (outcome-devaluation) values to guide goal-directed actions. We used a previously developed PIT task that allows distinguishing between specific and general PIT^24^, in which participants learn to liberate real snack foods from a virtual vending machine, followed by an outcome-devaluation phase to asses goal-directed control. Based on a theoretical framework of addiction^2^, we hypothesized that patients with AUD would show decreased goal-directed control and specific PIT effects, and would show increased habitual control and general PIT. We further hypothesized that addiction duration and severity would be negatively related to goal-directed control and specific transfer effects, and would be positively associated with habitual control and general transfer effects^2^. Following previous findings^20^, we expected increased NAcc activity during transfer to differentiate patients who would later relapse from abstainers.

## Methods

The study was approved by the Medical Ethics Committee of the Academic Medical Center, University of Amsterdam and performed in accordance with those guidelines. All subjects provided written informed consent. See Supplementary Methods for details on participant and exclusion criteria and study information.

### Participants

We recruited a total of 51 recently detoxified individuals with AUD and 32 healthy controls (HCs) for this study. HCs were recruited through advertisements and from our database. Individuals with AUD were recruited from a local addiction treatment center (Jellinek, Amsterdam) and were detoxified (>2 weeks), in treatment for and recently diagnosed with AUD without Axis 1 comorbidity. All subjects underwent the MINI structured psychiatric interview^27^, to confirm the absence of any psychiatric disorder (except for DSM-5 AUD in the AUD group).

Groups were matched on age, gender, BMI, years of education, handedness and intelligence quotient. AUD severity and alcohol craving were evaluated using a Dutch version of the Alcohol Use Disorder Identification Test (AUDIT, score range of 0–40)^26,28^ and the Dutch, five-item Obsessive-Compulsive Drinking Scale (OCDS, score range of 0–20)^27,29^. AUD patients indicated how long their alcohol use was problematic, reflecting chronicity of alcohol use. Lifetime alcohol intake was converted to kilograms pure alcohol by multiplying the total number of drinks (obtained using the Lifetime Drinking History questionnaire^30^) with 0.014 (kg pure alcohol in a standard drink). Six months following participation, individuals with AUD were contacted by telephone to assess relapse rates.

### Experimental design

**Figure 1:**
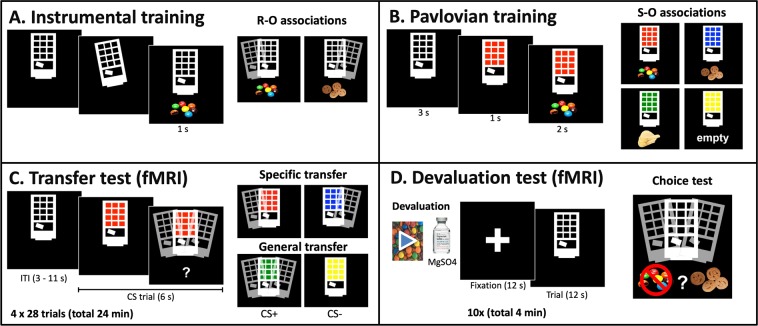
Overview of the four stages of the task and the contingencies. (**A**) Participants were able to tilt the vending machine to the left or right in order to obtain snacks. (**B**) During Pavlovian training, participants learned that specific light colors predicted specific outcomes. The transfer test (**C,D)** devaluation test were performed in the fMRI scanner during extinction (i.e. no outcomes were delivered). CS, conditioned stimulus; ITI, intertrial interval; fMRI, functional Magnetic Resonance Imaging.

We used a previously used Pavlovian-to-Instrumental Transfer (PIT) and outcome-devaluation task^24^. Participants were asked to abstain from eating for three hours before the start of participation. After providing consent, participants underwent ~1 hour of interviews, questionnaires and cognitive tests. Materials and procedure are described below shortly; for further details see Fig. 1 and Supplementary Materials. These data were collected as part of a more extensive study protocol including questionnaires, neuropsychological testing, another fMRI task, and a resting-state fMRI scan, data of which will be presented elsewhere.

#### Instrumental training

At the start of the experiment, participants tasted and rated all snacks. Participants were told they could steal snack foods from a virtual vending machine (Fig. 1A) and that they were allowed to eat the earned snacks at the end of the experiment. Participants were instructed to use two buttons to tilt the vending machine to the left or right, with each button press (R1 and R2) being associated with a different food snack (O1 and O2, respectively). Responses were reinforced on a variable ratio 7.5 schedule, meaning that on average every 7.5 repetitions (random number between 5 and 10) of one response would earn them a reward. When earned, snacks were shown on the screen and presented on a plate. Probe questions (“Which snack falls out when you tilt to the left/right”) tested explicit knowledge of the instrumental contingencies. Participants were trained until they reached a criterion of six consecutive correct answers on the probe questions testing explicit knowledge after every three snacks earned.

#### Pavlovian training

In the second phase, participants learned the predictive relationship between four different color lights of the vending machine and different snack foods (Fig. 1B). Two cues (S1 and S2) were associated with the same outcomes that were available during instrumental training (O1 and O2, respectively). Another cue (S3) was associated with a new snack (O3), i.e. CS+, while the fourth cue (S4) did not earn a snack food and the word “empty” was shown (CS−). After each block of four colored lights with corresponding outcomes, a multiple-choice question (“which snack will fall out”) was presented together with one of the colored lights to test knowledge of the Pavlovian contingencies. Subjects could use the keyboard [a b c d] to indicate their answer, after which feedback was provided. Pavlovian training ended after 12 blocks.

Participants were taken to the MRI scanner immediately following training, where they received two button boxes to tilt the vending machines using their right and left index finger. After making a structural scan, the instructions for the Pavlovian-Instrumental Transfer test started. The PIT and outcome-devaluation test phases took place while fMRI scans were acquired.

#### Pavlovian-Instrumental transfer test

This phase tested the influence of the four reward cues on instrumental responding, i.e., Pavlovian-to-instrumental transfer (Fig. 1C). Participants were told that they could again obtain snacks by tilting the machine. The vending machine was presented and during random intervals, reward cues were displayed. The four colored lights were presented 28 times each in pseudorandom order. Participants were able to tilt the machine both during cue presentation (6 sec) and during the intertrial interval (ITI) when the unlit vending machine (i.e. without colored lights) was presented (3–11 sec), thus employing an active baseline. Participants were told they could earn snacks, but no snacks were shown on the screen (i.e. extinction) to avoid contingent reinforcement.

Specific PIT is reflected by the specific influence of cues (S1 and S2) predicting food (O1 and O2, respectively) in biasing choice towards the action previously earning the same food (R1 and R2, respectively). General PIT is reflected by the general incentive motivational influence of the CS+ (S3) on responding, compared to the CS− (S4). The mean number of responses per trial (average duration = 6 sec) computed for each condition was used as the main outcome variable for the analyses. The PIT test comprised a total of 112 trials (28 trials/stimulus) with a total duration of ~24 minutes.

#### Outcome-devaluation

In this phase, one of the snacks (O1 or O2, counterbalanced) was devalued (see Fig. 1D). Following Morris *et al*. procedure^24^, this was initially done by presenting a video of the snack infested with waxworms. However, after testing 5 HCs we noticed that this manipulation was not effective and the procedure was altered during data collection (see supplement for more information). To make devaluation more robust, subjects additionally underwent a taste aversion procedure using a ﻿bitter solution of magnesium sulfate for the devalued snack^31^. This procedure, which took place outside the scanner, involved tasting the two snacks (O1 and O2), with the devalued snack containing a magnesium sulfate solution and being presented last. After entering the scanner again, participants watched a movie for 2 minutes with the devalued snack infested with waxworms. All participants included in the final analyses underwent the full procedure; the five participants (all HCs) from the full sample were not included in the outcome devaluation analyses because they only underwent the video-devaluation, which was ineffective.

To evaluate the effect of outcome-devaluation on action selection, participants were presented with the unlit vending machine (10 blocks of 12 seconds) and able to tilt the machine to earn snacks. A fixation cross (12 seconds) was presented before each block. This phase lasted ~4 minutes. After completing this choice test, participants were asked to rate each snack and their hunger on a 7-point Likert scale.

#### Explicit knowledge of instrumental and pavlovian contingencies

At the end of the outcome-devaluation, while still being in the scanner, participants were tested on their explicit knowledge of the instrumental and Pavlovian contingencies (e.g., “Which snack was associated with the red light/tilting to the left”).

### Magnetic resonance imaging data acquisition

All magnetic resonance imaging (MRI) was performed on a 3 Tesla, full-body Intera MRI scanner (Philips Medical Systems, Best, The Netherlands) equipped with a 32-channel phased array SENSE radiofrequency (RF) receiver head coil. Functional MRI scans were acquired using a T2*-weighted gradient multi-echo echoplanar imaging sequence with the following parameters: repetition time (TR) = 2375 ms; echo time (TE) = 9/26.4/43.8 ms; flip angle = 76°; field of view (FOV) = 224 × 121.8 × 224 mm; voxel size = 3 × 2.95 × 3 mm; matrix size = 76 × 73; slice thickness = 3 mm; slice gap = 0.3 mm; number of slices = 37, acquired in interleaved order. This multi-echo sequence was chosen for its improved BOLD sensitivity and lower susceptibility for artefacts, especially for ventral regions^32^. The first three scans were discarded to allow T1 saturation to reach equilibrium. A total of 637 and 102 volumes were acquired for the PIT and outcome-devaluation phases, respectively. A high-resolution T1-weighted structural image was acquired for each participant with the following parameters: voxel size = 1 × 1 × 1 mm; FOV = 236.679 × 180 × 256 mm; TR = 6.862 ms; TE = 3.14 ms, 150 slices, slice thickness = 1.2 mm, sampling matrix = 212 × 212 × 150, flip angle = 8°.

Participants entered the scanner in a head-first supine position and were able to view the screen (BOLDscreen 32 LCD, Cambridge Research Systems) using a mirror attached to the head coil, on which the task stimuli were presented. Participants were told they could use their left and right index fingers to tilt the machine using the two button boxes (CurrentDesigns).

### Behavioral analysis

Statistical analyses were conducted using SPSS Statistics version 22 (IBM Corp, Armonk, NY). A significance threshold of *p* < 0.05 (two-tailed) was used. Behavioral plots were made using GraphPad Prism, version 7.03 (La Jolla, CA, USA). Main results were tested using corresponding Bayesian analyses (JASP software, version 0.8.6)^31,33^, using JASP’s default priors. For results where *p* < 0.05, we report the BF_10_, which quantifies evidence in favor of the alternative hypothesis H1. For null-results (*p* > 0.05) we report the Bayes Factor BF_01_, which quantifies the relative evidence in favor of the null hypothesis. BF_01_ between 1 and three is considered to reflect anecdotal evidence in favor of the null hypothesis, BF_01_ > 3 reflects substantial support and values larger than 10 reflect strong support^34^.

Parametric independent samples t-tests were used to test between-group differences of single variables (e.g., demographic and clinical data, hunger scores) and Mann–Whitney U-tests in cases where the assumption of normality was violated. Mixed ANOVAs were used to test between-group differences of multiple variables (e.g., specific PIT, general PIT, outcome devaluation). Greenhouse-Geiser corrections were applied in case the assumption of sphericity was violated. Associations with clinical variables within AUD group were tested using Pearson correlations.

### fMRI analyses

#### Preprocessing

Imaging data were preprocessed using Statistical Parametric Mapping software (SPM12, Wellcome Trust Centre for Neuroimaging, London). Raw multi-echo fMRI data were combined into single volumes according to Poser *et al*.^32^: realignment parameters were estimated for the images acquired at the first echo time and consequently applied to images resulting from the two other echoes. Thirty volumes, acquired at the start of the PIT task during which a fixation cross was shown, were used to calculate the optimal weighting of echo times for each voxel by applying a PAID-weight algorithm^32^. The multi-echo fMRI data were then combined into single volumes using these weightings. All functional images were subsequently slice-time corrected and co-registered with the high-resolution T1-weighted image using normalized mutual information. The high-resolution structural scan was segmented and used to normalize the slice-time corrected functional images. Finally, all functional images were smoothed with an 8 mm isotropic full-width at half maximum (FWHM) Gaussian smoothing kernel. In all first-level models, six movement regressors were included as regressors of no interest to account for translation and rotation variability. Regressors were convolved with the canonical hemodynamic response function and a high-pass filter with a cut-off of 128 seconds was applied to remove drifts within sessions.

#### Transfer test

A general linear model (GLM) was constructed for each participant: individual regressors were made for specific PIT (S1 and S2 combined), CS+ (S3), CS− (S4) and inter-trial interval trials (ITI; active baseline), modeled at trial onset as boxcar (6 seconds) functions. These were used for the between-subject PIT effects (see below). Similar to previous PIT-fMRI studies^23,24^, each trial was additionally parametrically modulated with the number of responses during that trial. These regressors were used for the within-subject contrasts. An additional regressor of no interest was constructed to account for variance induced by motor responses, modeling all key-presses as a stick function^29,35^. We used an active baseline (as opposed to a passive/implicit baseline) because it contains all the features of the CS trials (including movement, button presses, visual features), which decreases concerns with motor responses.

Following Talmi *et al*.^35^, PIT effects were identified on two levels: between- and within-subject. To assess individual (‘between-subject’) differences in the strength of the PIT effect, global PIT rates (specific PIT = total same minus different responses; general PIT = total CS+ minus CS− responses) were used at the second-level analysis as a covariate of interest. The first-level contrast images that were entered into the second-level analyses were [specific PIT > ITI] for the specific PIT effect and [CS+ > CS−] for the general PIT effect. These contrasts capture individual variation in the strength of the PIT effects.

For the within-subject specific PIT effect, the number of responses for the ‘same’ outcome during each trial was used as parametric modulator and compared to the ITI parametrically modulated by the number of responses. This contrast thus reveals BOLD activity that correlates with the influence of reward cues on choice on a trial-by-trial basis. Within-subject general PIT was identified by comparing CS+ trials parametrically modulated by the number of responses during each trial with CS− trials to reveal neural activity correlated with a general incentive effect of reward cues. First level activity maps were then entered into second-level two-sample t-tests to compare the AUD group with the HC group. These contrasts capture the trial-by-trial variation in the strength of the PIT effects ‘within’ each subject.

#### Devaluation test

The first level GLM for the devaluation test included two response regressors (valued and devalued) modeling each response as stick-functions, and one regressor modeling the ten blocks as 12-sec boxcar functions. A first level contrast was made comparing valued and devalued responses to identify neural activity related to the new action values after devaluation. Participants who made no devalued responses were excluded from the fMRI analysis as responses are needed to fit the regressor to the timecourse. Additionally, participants were excluded if they made <5 devalued responses, which was chosen because it conforms with the minimal number of responses made by participants in a previous fMRI PIT study^23^.

#### Region of interest analyses

Region of interest (ROI) analyses were performed using masks used previously^24^ and included for PIT effects: the bilateral amygdala, bilateral putamen, the ventral striatum, and the medial OFC; for outcome-devaluation: the medial OFC, medial PFC and the bilateral caudate. Following Garbusow *et al*.^20^, additional ROI analyses were performed by extracting the mean parameter estimates from the individual contrast images for the right and the left NAcc. See Supplement for details.

### AUD severity, chronicity, and relapse

We also examined whether the ability of reward-related cues to guide actions was associated with (i) the amount of alcohol used and (ii) the severity of alcohol-related problems in individuals with AUD and (iii) duration of abstinence. Using Spearman correlations, we tested the relationship of specific PIT, general PIT, and outcome-devaluation with (i) lifetime alcohol intake (in kg), (ii) AUDIT scores and OCDS scores and (iii) number of weeks participants were abstinent. Moreover, we investigated the relationship of these three factors with neural responses for the within-subject PIT effects and outcome-devaluation by including them as a covariate (tested for each factor separately) in the relevant fMRI analyses. To test the association of between-subject PIT effects with chronicity, severity and abstinence, individual parameter estimates were extracted from the bilateral NAcc ROIs. These were used in several multiple regression analyses (forced entry) to predict the left/right NAcc beta values from the specific/general PIT contrasts based on the respective global PIT rates (first predictor) and the severity/chronicity/abstinence measures (second predictor). We additionally explored whether the behavioral and neural PIT and devaluation effects would be predictive of relapse at follow-up^20^. Non-significant results were complemented by Bayesian linear regression analyses.

## Results

The test of explicit knowledge about instrumental and Pavlovian contingencies at the end of the experiment revealed that 13 individuals with AUD and 10 HCs did not retain all contingencies correctly. Because this knowledge is crucial for the interpretation of the PIT and devaluation tests and following previous research^35^, these participants were excluded from all analyses (see Supplementary Figs. 7–9). Note, however, that including these participants in the analyses does not actually change the pattern of results (main effects and group comparisons; analyses not reported). Demographic and clinical characteristics of the remaining 38 individuals with AUD and 22 HC participants are summarized in Table 1.

**Table 1:** Demographic and clinical results, Mean (SD).

	AUD (n = 38) Mean (SD)	HC (n = 22) Mean (SD)	*p* value
Age	44.4 (11.2)	43.0 (10.6)	0.64
Male (%)	27 (71%)	17 (77%)	0.60^a^
BMI	25.7 (4.3)	23.1 (2.8)	**0.02**
Education, years	10.5 (3.1)	10.2 (3.7)	0.88
IQ	101.8 (14.2)	98.3 (9.6)	0.32
Digit span	17.5 (4.1)	17.7 (4.7)	0.84
Smokers (%)	23 (61%)	3 (14%)	**<0.001**^a^
AUDIT	24.7 (6.1)	3.5 (2.1)	**<0.001**^b^
OCDS-D	4.4 (2.8)	1.0 (1.9)	**<0.001**^b^
Lifetime alcohol intake (kg pure alcohol)	496.3 (394.2)	53.0 (55.3)	**<0.001**
Weeks abstinent	7.1 (6.6)	—	—

AUD: alcohol use disordered patients; HCs: Healthy Controls; SD: Standard Deviation; BMI: Body Mass Index; IQ: Intelligence Quotient; AUDIT: Alcohol Use Disorders Identification Test; OCDS-D: five-item Obsessive Compulsive Drinking Scale – Dutch; ^a^p value of chi-square test, ^b^p value of Mann-Whitney test, all other represent p values of two-sampled t-test.

### Behavioral pavlovian-instrumental transfer results

#### Food and hunger ratings across groups before training

Mean subjective ratings for the snack foods and hunger were positive (>4) in both groups. Both groups rated the three snacks (O1, O2, O3) and their hunger similarly, as revealed by independent samples t-tests (p > 0.05).

#### Instrumental Conditioning

Acquisition of the instrumental contingencies was similar across groups: the number of snacks earned for both outcomes and both responses made were similar between groups (all p > 0.1; see Supplementary Materials), indicating that both groups successfully learned the response-outcome contingencies.

#### Pavlovian conditioning

The average number of probe questions that were answered correctly (HC = 11.3, ±1.5; AUD = 11.7, ±0.6) did not significantly differ between the groups (t_58_ = 1.23, p = 0.22), indicating that all groups learned the stimulus-outcome contingencies.

#### Specific PIT

**Figure 2:**
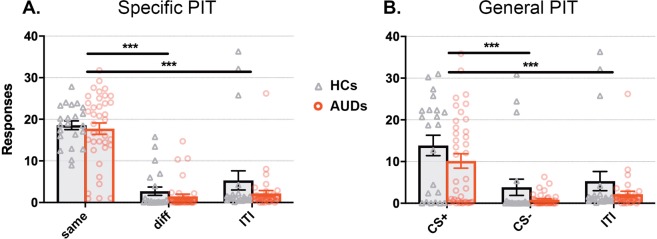
Strong specific and general PIT effects in both AUD patients and HCs. (**A**) Reward cues increased choices for the same outcome in both groups compared to different responses and responses during the ITI (i.e., specific transfer). (**B**) In both groups, the reward cue (CS+) increased responding significantly more than the non-reward cue (CS−) and ITI, indicating general transfer. ***Main effect of condition p < 0.001.

A main effect of response-type (F_1.63,94.2_ = 109.74; p < 0.001, η^2^ = 0.65) indicated a highly significant specific PIT effect (Fig. 2A): the action that delivered the same outcome as that predicted by the stimulus (‘same’) was chosen significantly more than the other action (‘diff’: t_58_ = 13.56, p < 0.001, d = 1.75) and the active baseline (‘ITI’: t_58_ = 10.98, p < 0.001, d = 1.42). Contrary to our hypothesis, this specific PIT effect did not differ between the groups as revealed by the absence of a group x response-type interaction (F_1.63,94.2_ = 0.52; p = 0.60, η^2^ = 0.003), nor was there an overall difference in responding between groups (main effect of group: F_1,58_ = 2.81; p = 0.10, η^2^ = 0.05, BF_01_ = 3.25). The Bayesian mixed ANOVA further revealed substantial evidence (BF_01_ = 6.25) in favor of the null hypothesis that groups did not differ on specific PIT (group x response-type interaction).

Within individuals with AUD, no significant associations were found between specific PIT (same minus different responses) and alcohol-use chronicity and AUD severity measures (all p > 0.12; r < 0.26). Bayesian correlations showed substantial evidence for the absence of a relationship between the specific PIT effect and lifetime alcohol intake and duration of abstinence (BF_01_ = 4.74, BF_01_ = 3.99), and anecdotal evidence against a relation with OCDS (i.e. compulsive drinking; BF_01_ = 2.86) and AUDIT (i.e. severity of drinking problems; BF_01_ = 1.84).

#### General PIT

As can be seen in Fig. 2B, there was a significant general PIT effect across both groups (main effect of cue-type: F_1.25,65.3_ = 36.88; p < 0.001, η^2^ = 0.39): the CS+ induced general incentive motivation relative to the CS− (t_58_ = 7.04, p < 0.001, d = 0.91) and ITI (t_58_ = 5.73, p < 0.001, d = 0.74). There was no significant main effect of group (F_1,58_ = 3.61; p = 0.06, η^2^ = 0.06, BF_01_ = 1.04) or a group by cue-type interaction (F_1.25,65.3_ = 0.05; p = 0.85 η^2^ = 0.001). The BF_01_ for an interaction between group and trial type was 9.71, suggesting substantial to strong evidence in favor of the null hypothesis that the general PIT effect did not differ between groups. The number of button-presses during the active baseline (ITI) did not significantly differ between the groups (p = 0.21).

We found no significant correlations of general PIT (CS+ minus CS− responses) with chronicity, severity and abstinence measures (lowest p = 0.48). Bayesian evidence against a relationship between these factors and general PIT was substantial (BF_01_ 3.90–4.94).

Number of responses (within and across trials) did not differ between groups for either the specific nor general transfer effect (sFigs. 4 and 5).

### Neuroimaging pavlovian-instrumental transfer results

One AUD patient was excluded from the PIT fMRI analysis due to excessive movement (>3 mm translation), while none were excluded due to rotation (all <0.07°). Two individuals with AUD did not show any variation in the number of responses during the PIT phase (e.g. always one), while three subjects (2 AUD, 1 HC) did not show any variation only for general PIT trials. As this renders parametric modulation analyses impossible these subjects were excluded from the ‘within-subject’ analyses.

All main results referred to in this section are available online at https://neurovault.org/collections/ETZNNLKP/. See Table 2 for peak coordinates of the main contrasts.

**Table 2:** fMRI results across all participants (AUD and HC).

	Region	L/R	X	Y	Z	k	FWE *p*	*t* value	Z
**Specific PIT effect** (between-subject), within ROI mask	Caudate	R	9	11	−1	31	0.009	5.46	4.86
	Putamen^a^	R	33	−7	−4	70	0.009	5.46	4.86
	Putamen^a^	R	27	2	−1		0.027	5.10	4.59
**General PIT** (between-subject), within ROI mask	Putamen	R	33	−1	−1	79	0.000	7.41	6.14
	Pallidum	R	24	−1	−1	21	0.000	7.32	6.09
	Putamen	L/R	−30	−10	−1	56	0.047	4.91	4.45
**General PIT** (within-subject), whole-brain	mOFC	R	12	38	−10	28	0.001	6.16	5.32
		L	−12	38	−10	22	0.005	5.71	5.01
	ACC	R	12	35	2	2	0.028	5.17	4.62

All p-values peak-level FWE-corrected. mOFC = medial OrbitoFrontal Cortex; ACC = Anterior Cingulate Cortex, ^a^extends to right pallidum. X, Y and Z coordinates are reported in MNI space.

#### Specific PIT

**Figure 3:**
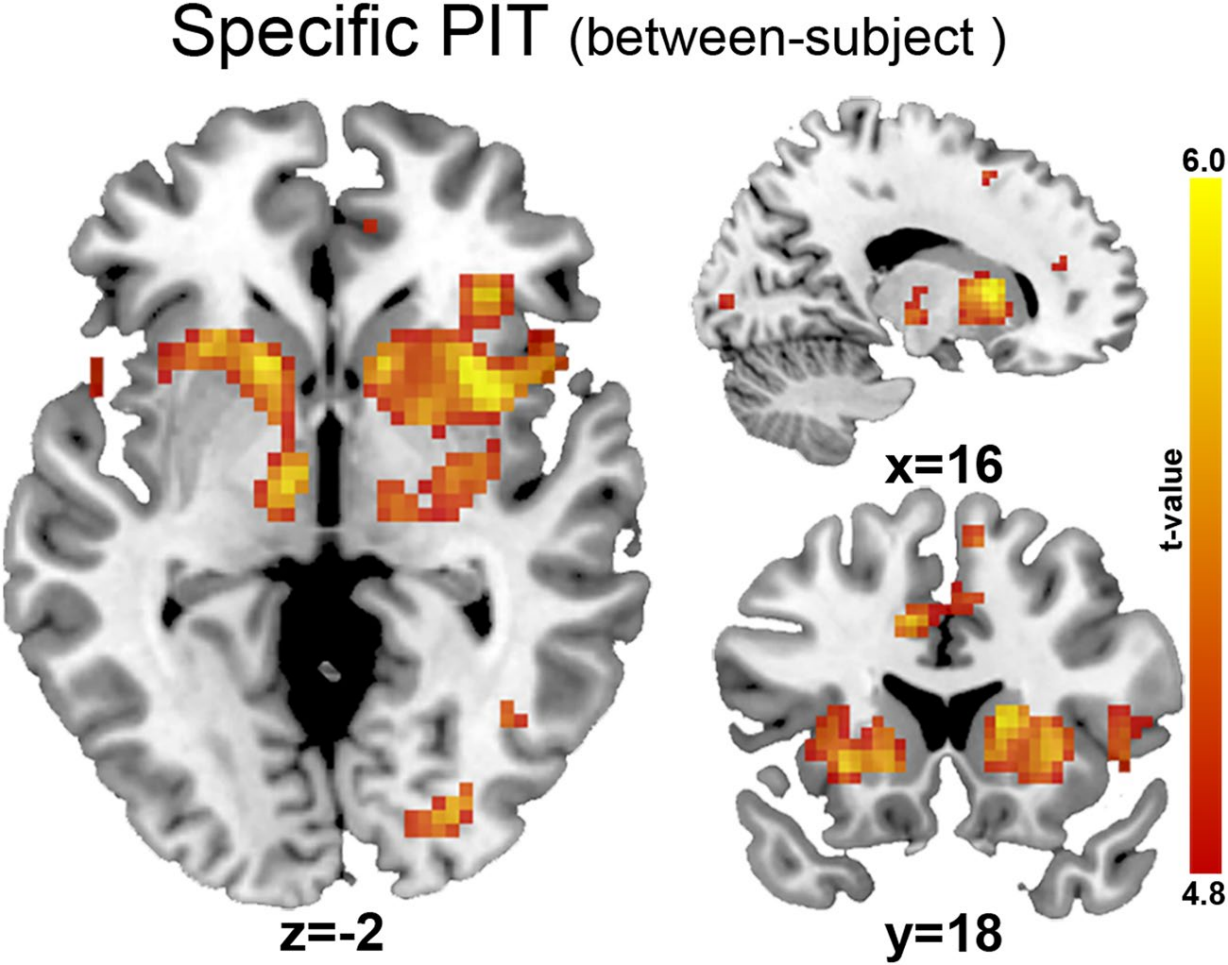
Between-subject specific PIT effect across groups, whole-brain (p < 0.05 FWE-corrected). No differences between AUD patients and HCs were found.

Whole-brain analyses across groups revealed that specific PIT (between-subject) was associated with neural activity in regions including the bilateral caudate, putamen, thalamus, pallidum, hippocampus, insula, middle cingulate, supplementary motor area extending towards the medial frontal superior gyrus; and the left medial and right posterior OFC (p_FWE_ < 0.05 voxel-level; Fig. 3). When masked with the PIT ROI, this analysis further confirmed the involvement of the bilateral putamen, caudate, thalamus, pallidum and insula. No regions were significantly related to within-subject specific PIT. However, no significant differences in the neural patterns associated with specific PIT were observed between the individuals with AUD and HCs.

Within the AUD group, we found no significant whole-brain association between within-subject specific PIT and chronicity or severity measures. Multiple ROI regression analyses revealed no significant effects of chronicity, severity or abstinence on between-subject specific PIT in the left NAcc (total lifetime alcohol intake: p = 0.77, BF_01_ = 2.18; AUDIT: p = 0.95, BF_01_ = 2.24; OCDS: p = 0.46, BF_01_ = 1.82; weeks abstinence: p = 0.80, BF_01_ = 2.19) or right NAcc (total lifetime alcohol intake: p = 0.71, BF_01_ = 2.41; AUDIT: p = 0.37, BF_01_ = 1.86; OCDS: p = 0.79, BF_01_ = 2.48; weeks abstinence: p = 0.93, BF_01_ = 2.54).

#### General PIT

**Figure 4:**
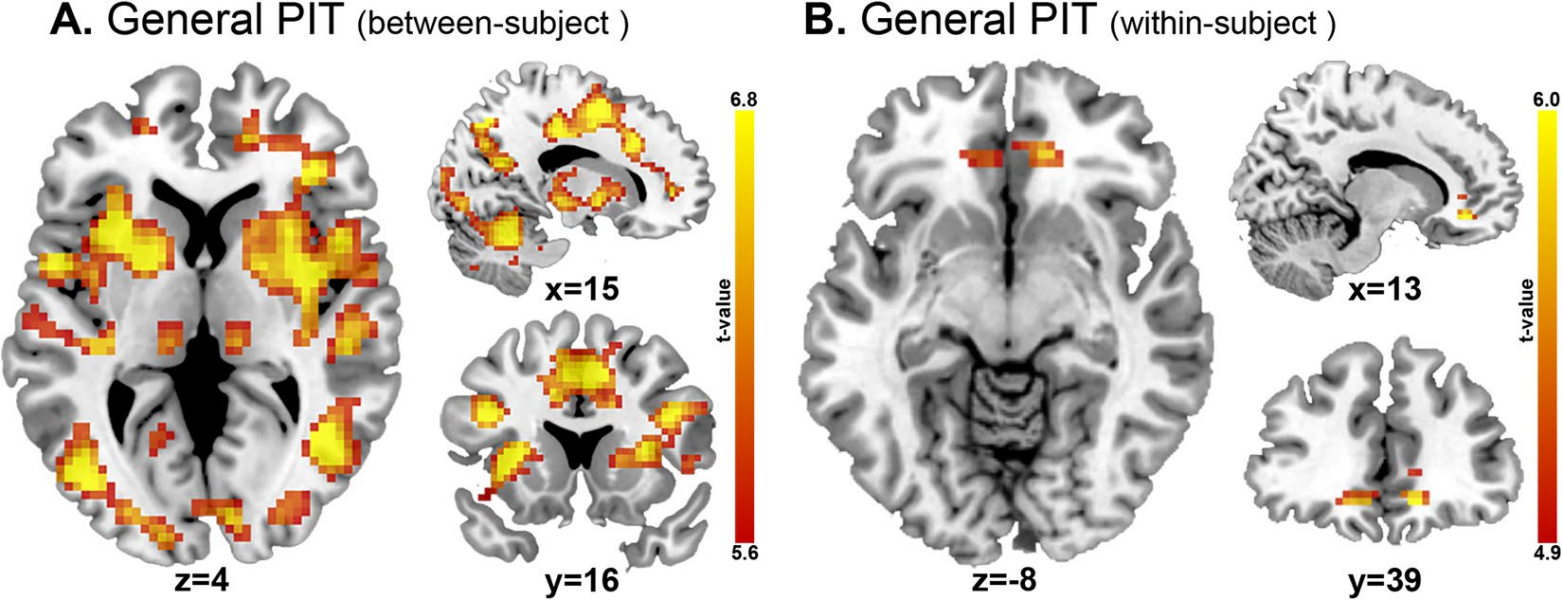
General PIT effect. (**A**) Between-subject general PIT effect across groups, whole-brain (p < 0.05 FWE-corrected). No differences between AUD patients and HCs were found. (**B**) Within-subject general PIT effect across groups, whole-brain (p < 0.05 FWE-corrected). No differences between AUD patients and HCs were found.

The incentive effect of the CS+ on responding (compared to CS−) was associated with activity in several large clusters (Fig. 4A), including the bilateral putamen, ACC, insula, SMA, thalamus; the right amygdala; and the left hippocampus (whole-brain p_FWE_ = 0.05 voxel-level). ROI analyses of this between-subject general PIT contrast revealed activations in the bilateral putamen, caudate, thalamus, pallidum and insula (p_FWE_ = 0.05 voxel-level). Across both groups, within-subject general PIT was associated with increased activity in bilateral medial OFC and left ACC (both in the whole-brain and ROI analyses; Fig. 4B). However, no significant differences between the individuals with AUD and HCs were observed.

Within the AUD group, whole-brain analyses of the within-subject general PIT effect indicated that no regions were significantly associated with chronicity or severity measures. Moreover, multiple regression ROI analyses revealed no significant effects of chronicity, severity or abstinence on general PIT (between-subject) in the left NAcc (lifetime alcohol intake: p = 0.54, BF_01_ = 2.36; AUDIT: p = 0.63, BF_01_ = 2.49; OCDS: p = 0.98, BF_01_ = 2.73; weeks abstinence: p = 0.54, BF_01_ = 2.65) or right NAcc (lifetime alcohol intake: p = 0.62, BF_01_ = 2.76; AUDIT: p = 0.51, BF_01_ = 2.56; OCDS: p = 0.45, BF_01_ = 2.42; weeks abstinence: p = 0.89, BF_01_ = 3.04).

### Behavioral outcome-devaluation results

#### Subjective ratings after outcome-devaluation

**Figure 5:**
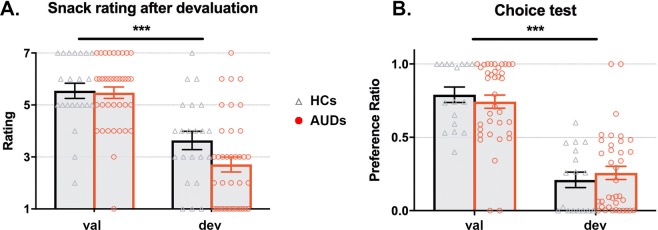
The outcome-devaluation procedure successfully decreased subjective rating (**A**) and responding (**B**) for the devalued snack across groups. (**A**) Subjects in both groups rated the devalued snack significantly lower than the valued snack, indicating devaluation was successful. (**B**) The proportion of choices for the devalued snack was significantly reduced after the devaluation procedure in both groups, as seen here by the preference ratio for valued versus devalued responses, indicating goal-directed control. ***Main effect of devaluation, p < 0.001.

After the devaluation procedure, mean snack ratings for the devalued snacks were negative (<4) while still being positive for the valued snacks (>4) across the remaining 38 AUDs and 17 HCs (Fig. 5A). This main effect of devaluation was significant (F_1,53_ = 54.40; p < 0.001, η^2^ = 0.51) indicating that the devaluation procedure was successful, but did not differ between the groups (no main-effect of group: F_1,53_ = 0.44; p = 0.51, η^2^ = 0.01, BF_01_ = 3.89; or group x type interaction: F_1,53_ = 0.95; p = 0.33, η^2^ = 0.02, BF_01_ = 1.91). Hunger ratings were similar between groups after the experiment (t_58_ = 0.36; p = 0.72, d = 0.01,, BF_01_ = 3.51).

#### Instrumental outcome-devaluation: choice test

During the choice test following the devaluation procedure, the number of responses was significantly decreased for the devalued relative to the valued snack (main effect of response-type: F_1,53_ = 54.26; p < 0.001, η^2^ = 0.51), indicating goal-directed control of action selection in both groups (sFig. 3 and Fig. 5B). However, no significant effect of group (F_1,53_ < 0.001; p = 0.99, η^2^ < 0.001) or an interaction (F_1,53_ = 0.01; p = 0.92, η^2^ < 0.001) was found. The Bayesian analysis further substantiated the absence of a group difference, offering substantial evidence (BF_01_ = 3.61) in favor of the null hypothesis.

No significant correlations between alcohol use chronicity, abstinence duration or severity measures with outcome-devaluation were found (all p > 0.05). Bayesian evidence in support of the null hypothesis was substantial for the AUDIT (BF_01_ = 3.88) and duration of abstinence (BF_01_ = 4.82), but inconclusive for the relation with lifetime alcohol intake (BF_01_ = 0.99) or OCDS (BF_01_ = 0.85).

### Neuroimaging Outcome-devaluation results

We excluded eight individuals with AUD and five HCs from the fMRI analysis because they made zero devalued responses and another two individuals with AUD and two HCs because they made <5 devalued responses, resulting in 29 individuals with AUD and 10 HCs for this analysis. The fMRI outcome-devaluation analysis revealed no significant activations across and between the groups, nor any significant association with alcohol use chronicity or severity of AUD problems.

### AUD relapse

Out of the 38 individuals with AUD that were included in the final analyses, 22 remained abstinent after six months (58%), 12 relapsed (32%) and four could not be reached (10%). We assessed whether any of the task-related variables would be predictive of relapse by comparing relapsers and abstainers, but did not find significant behavioral differences on specific PIT (F_1,32_ = 1.02; p = 0.32; BF_01_ = 3.39), general PIT (F_1,32_ = 0.06; p = 0.81; BF_01_ = 3.33) or outcome-devaluation (t_32_ = 0.53, p = 0.58; BF_01_ = 2.64).

The group comparison between the relapsers and abstainers revealed no differences in neural patterns on general or specific PIT or outcome-devaluation. Moreover, relapsers and abstainers did not significantly differ in NAcc activity during PIT, as revealed by independent-samples t-tests for within-subject general PIT (left NAcc: p = 0.90, BF_01_ = 2.79; right NAcc: p = 0.42, BF_01_ = 2.19) and specific PIT (left NAcc: p = 0.21, BF_01_ = 1.53; right NAcc: p = 0.154, BF_01_ = 1.29); and by multiple regression analyses for between-subject general PIT (left NAcc: p = 0.78, BF_01_ = 2.39; right NAcc: p = 0.85, BF_01_ = 2.81) and specific PIT (left NAcc: p = 0.52, BF_01_ = 1.86; right NAcc: p = 0.57, BF_01_ = 2.05).

## Discussion

In this fMRI study, we investigated whether abstinent, in-treatment individuals diagnosed with AUD suffer from impairments in associative learning and decision-making, specifically deficits in goal-directed control over action and the influence of Pavlovian, predictive cues on instrumental choices (i.e. PIT). Contrary to our hypotheses, individuals with AUD did not show any deficits relative to healthy controls in the ability to use outcome knowledge to guide choice, or in specific or general PIT. This was further reflected by the absence of significant differences between AUD and HC groups in neural activity underlying goal-directed actions and PIT. Furthermore, we tested within the AUD group whether prior chronicity of alcohol use or severity of alcohol-related problems were related to deficits in associative learning and decision-making processes but again found no significant associations. All main null findings were supported by substantial Bayesian evidence, although the evidence against associations with clinical measures was not always decisive.

In both HC and individuals with AUD, we observed strong behavioral general and specific PIT effects. The brain areas that were associated with these behavioral effects converge with previous findings in both animals and humans. Both general and specific PIT were mediated by activity in the amygdala^21,23,35,36^, the bilateral pallidum^37,38^ and various subregions of the striatum, including the bilateral putamen, caudate and thalamus^23,35,38^. Finally, we observed that responses induced by increased incentive motivation during general PIT were related to increased mOFC activity. Although the OFC has mostly been implicated in specific transfer in animal studies^13^, our findings replicate previous activation patterns found in healthy subjects during general PIT^24^. Overall, the behavioral and neural effects observed in our study closely resemble previous findings in healthy populations^23,35,36,38,39^, indicating that the PIT paradigm produced robust and reliable transfer effects across groups.

However, inconsistent with our expectations, we found no behavioral or corticostriatal deficits in specific or general PIT in subjects in treatment for AUD. Bayes factors provided substantial evidence for an absence of behavioral group differences. Few studies have so far investigated PIT in alcohol users. Two studies assessed specific PIT in non-clinical alcohol users but failed to find any relation with severity of alcohol use^40,41^. One previous neuroimaging study in detoxified individuals with AUD investigated the effect of both non-alcohol^20^ and alcohol-related stimuli^42^ on instrumental responding. In this PIT task, monetary gains and losses were used as rewards, which might differ from the primary food rewards used in our study. Alcohol-related stimuli had an inhibitory effect on responding in individuals with AUD, accompanied by increased NAcc activity^42^. This conflicts with the expected positive motivational effect of alcohol-related stimuli that has previously been found in rats^43^, but may be a consequence of the negative properties that alcohol stimuli gained during abstinence. Non-alcohol stimuli, on the other hand, had a more pronounced effect on responding in patients than HCs, but (similar to our findings) no PIT-related differences in BOLD response were observed. Moreover, NAcc activity was found to be higher in the subgroup of AUD patients that relapsed (n = 13) compared to the abstainers (n = 11)^20^, a finding we could not replicate in our similarly small sample (12 relapsers and 22 abstainers). In a larger sample of participants, relapsers were found to show stronger PIT effects behaviorally on the same task^44^, as was the case for the highly impulsive AUD patients in this sample^45^. Importantly, these stronger PIT effects were specifically driven by patients being less able to overcome conflict, i.e. when the Pavlovian cue predicts an outcome (e.g. a win) that is incongruent with the required action (e.g. to avoid), indicating inhibition problems. Congruent PIT effects, i.e. when Pavlovian-paired stimuli enhance responding for valuable outcomes, did not significantly differ in individuals with AUD compared to controls. As this is similar to the way in which PIT was probed in our study, these results do not conflict with our findings.

Contrary to the predicted deficits of goal-directed control in AUD, the results on the choice test following outcome-devaluation suggest an intact ability of individuals with AUD to modify action selection following changes in outcome value. Bayesian factors provided substantial evidence for the null hypothesis, indicating that individuals with AUD show no impairments integrating changes in experienced value with the action-outcome association. Although our findings are not in line with studies in animals^46^, evidence in humans is less convincing, as critically reviewed recently^47,48^. Animal studies have mostly relied on simple lever-press procedures to reveal impaired goal-directed control after chronic drug-exposure, which may produce stimulus-response “drug-habits” by design: recent evidence suggests that addiction-like behavior can emerge even when rats have to solve complex puzzles, which prevents drug seeking to become habitual, and that drug seeking remained under ventral striatal control^49^. Outcome-devaluation paradigms in human drug users have provided mixed evidence for impaired performance. Previous studies found impaired performance on an outcome-devaluation task in alcohol^12^ and cocaine dependence^50^, while several studies failed to find any impairment in tobacco dependence^51–53^. A recent paper in treatment-seeking drug users provided further evidence for intact goal-directed control, on outcome-devaluation and specific PIT tasks, across two experiments^47^. An additional goal of the current study was to investigate the relation between PIT and outcome-devaluation strength with various AUD-related measures. Again, however, we did not find any associations between AUD severity and the expression of PIT or devaluation, which was further supported by substantial Bayesian evidence in most cases.

To summarize, the current findings converge with previous research to suggest intact general (i.e. unrelated to the addiction) associative learning mechanisms in abstinent AUD patients. Future studies will have to determine whether these processes are intact when it comes to (i) active alcohol users, (ii) alcohol-related cues and (iii) alcohol-seeking behavior. These issues are important because, although we found no association between duration of abstinence with PIT and outcome devaluation strength, decision-making deficits related to substance-seeking may still be impaired in active users. Behavior in patients with AUD may be strongly driven by alcohol-cues that have a disruptive influence on decision-making, hindering goal-directed behavior. In line with this idea and mirroring findings in rodents^43^, one study showed that goal-directed control of cigarette seeking in smokers was abolished when participants were situated in a bar lab and presented with an alcoholic beverage^54^. In sum, although our results provide evidence for an intact system regarding newly learned associations and food rewards, future studies should determine the influence of naturalistic contextual cues on alcohol-seeking behaviors and the extent to which these are under habitual control in AUD patients.

There are several limitations to our study. First, the number of smokers was significantly higher in the AUD group, which may have affected the (absence of) described effects. Further, we cannot exclude the possibility that subjects already explicitly learned the shared outcome of the instrumental and Pavlovian contingencies during Pavlovian training. Such an explicit association may have led to more declarative tactics during the transfer phase and as a consequence may have obscured group differences. Unfortunately, there is no way to assess this in the current dataset, as the Pavlovian condition took place in the absence of any behavioral component and outside the scanner. No significant neural activations were seen when comparing devalued and valued choices on the outcome-devaluation paradigm, possibly as a result of the low number of devalued responses resulting in low power. Additionally, due to signal dropout especially in the ventral part of the OFC, we are unable to draw strong conclusions about this region’s involvement in control of goal-directed action and potential group differences.

To conclude, this study was the first to dissociate between specific and general PIT and their neural correlates in human AUD, using food rewards. Our results provide substantial evidence that human individuals with AUD are not impaired in general goal-directed or cue-guided choice behavior, or the neural circuits mediating these processes. Based on the results presented in the current paper and the limited evidence for impaired goal-directed and habitual control in AUD, it may be premature to rely heavily on the theory that general associative learning processes are disrupted in human AUD. This conclusion seems, however, in conflict with a bulk of animal literature on PIT^6,46^. This discrepancy may be related to differences between human and animal studies, in which the latter generally test the influence of drug-related environments on drug seeking in actively using animals. Future studies may investigate the role of associative learning directly related to drug cues and drug seeking in active users, to better understand how such processes contribute to the development and maintenance of and relapse to substance use disorders^55^.

## Supplementary information

Supplementary Information.

Dataset1.

## Data Availability

The behavioral datasets analyzed during this study are available online in the Supplementary Data file. All main fMRI results are available online at https://neurovault.org/collections/ETZNNLKP/. Raw behavioral data and individual first-level t-maps are available from the corresponding author on reasonable request.
