# Supplementary material for: Intact corticostriatal control of goal-directed action in Alcohol Use Disorder: a Pavlovian-to-instrumental transfer and outcome-devaluation study
Source: Sci Rep. 2020 Mar 18;10:4949. doi: 10.1038/s41598-020-61892-5 (PMC7087408; doi:10.1038/s41598-020-61892-5)
Supplement: Supplementary file 1 — Supplementary Information. [file 41598_2020_61892_MOESM1_ESM.docx]

**Supplementary information**

Intact corticostriatal control of goal-directed action in Alcohol Use Disorder: a Pavlovian-to-instrumental transfer and outcome-devaluation study

Tim van Timmeren^*^, Stephanie L. Quail, Bernard W. Balleine, Dirk E.M. Geurts, Anna E. Goudriaan^#^, Ruth J. van Holst^#^

**Supplementary Methods**

***Participants***

All subjects were aged between 18 and 65 years and included between December 2015 and June 2017.

Initially, our study was designed to include and compare a non-chronic (<2 years AUD history; <2 treatments) and chronic (>7 years AUD history; >3 treatments) AUD group. However, recruiting patients meeting these exact (and arbitrary) cut-offs for AUD history and number of treatments was untenable. Therefore, the AUD criteria were changed during the study to include a broad range in years of AUD history, number of treatments and lifetime alcohol intake, while still including ~twice as many AUD patients to do the correlational analyses. This design facilitated the investigation of chronicity by looking at severity and duration of AUD within the patient group.

From the full sample of contacted AUD patients, n=15 were labeled as relapsers (33%), while n=30 were labeled as abstainers (66%) and six AUD patients could not be reached.

**Exclusion criteria** included: lifetime history of bipolar disorder, anxiety disorder, obsessive-compulsive disorder or schizophrenia; past 6-month history of major depressive episode; current or past-year substance use disorder or current psychiatric treatment (except for AUD in the AUD patient group); the use of any psychotropic medication; positive breathalyzer test (alcohol) or urinalysis (benzodiazepines, (meth)amphetamines, opioids, cocaine, ecstasy, PCP, methadone or cannabis); history or current treatment for neurological disorders; major physical disorders; history of brain trauma; or any contraindications for MRI. In addition, HCs were excluded if they scored higher than seven on the Alcohol Use Disorder Identification Test (Conigrave, Hall, & Saunders, 1995). One AUD patient had a positive urine screen for MDMA; the subject declared that use was >1 week ago and he did not use regularly. A psychiatric interview further verified the absence of a (history of) dependence, and the subject was included in the analyses. Additional analyses indicated that the data of this individual fall within one SD of the mean of the AUD group for all main (behavioral and ROI) effects of interest. Note that this also means that these values do not diverge from the mean values of the HCs, as indicated by the Bayes Factors reported in the main text.

Verbal IQ was estimated by the Dutch Adult Reading Test (Schmand, Bakker, Saan, & Louman, 1991). The digit span (part of the Wechsler Adult Intelligence Scale; score range of 0-24) was used to assess general information processing speed (Wechsler, 1981).

***Task***

We used the same Pavlovian-to-Instrumental Transfer (PIT) and outcome-devaluation task Morris et al. (Morris, Quail, Griffiths, Green, & Balleine, 2015). All instructions were translated to Dutch. The task was programmed and run in PsychoPy v1.85.6 (Peirce, 2007), using a Windows computer with a 27-inch screen.

First, participants tasted six snacks: M&Ms, Speculaas cookies (‘Smoeltjes’), licorice candy (‘drop’), Pringles (Original), salted peanuts and rosemary flavored crisps. They were asked to rate all snacks on a 7-point Likert scale. The three highest-rated snacks were then selected as rewards in the experimental paradigm. Participants were subsequently seated behind a computer in a quiet room near the MRI scanner.

*Instrumental training*

The following instructions were presented: “Someone has said you can get free snacks from our vending machine. Use the keyboard to tilt the machine to the left and right, and learn to steal different snacks!” Participants were instructed to use the keys ‘f’ (left) and ‘j’ (right) on a keyboard to tilt the vending machine.

*Pavlovian training*

Before Pavlovian training started, new instructions were presented: “We have discovered that people were stealing from the vending machines! We have upgraded the machine to make them harder to steal from. However, when the machine is overstocked a free snack will fall out. The lights on the front panel indicate whether the machine is overstocked. Watch the lights and learn which snacks fall out.”

The next two phases of the experiment were done in the MRI scanner.

*Pavlovian-Instrumental Transfer (PIT) test*

Participants received the following instructions: “You have now found one of the new, improved vending machines. At random intervals the machine will be overstocked with one of the snacks. You can see this again by the color of the lights on the front. You can tilt the machine to get the snacks. However, you will no longer receive feedback about the snacks, so no snacks will appear on the screen. Remember what you have learned before, the associations are the same. We will keep track of the amount of snacks you deserve to eat later. You will hear how much you have earned afterwards. Try to get as many snacks as you want!”

*Devaluation test*

We initially used a devaluation procedure similar to Morris et al. (2015), using only a devaluation video. However, this turned out not to be effective, as participants did not change their snack ratings of the devalued snack, nor did it affect responses made on the choice test. Therefore, we added a taste aversion procedure using a magnesium sulfate solution. Because the devaluation procedure took place after the PIT test, we excluded data from the five HCs who did not receive the taste aversion procedure only from the analyses pertaining to the devaluation phase. All subjects included in the final outcome-devaluation analysis were thus presented a video of the snack infested with waxworms before undergoing taste aversion for one of the snacks.

All participants included in the analyses were temporally removed from the scanner to taste the snacks that were associated with an instrumental action (O1 and O2). One of the snacks (counterbalanced) was devalued by inducing taste aversion using a magnesium sulfate solution. Participants were told that the taste of one of the snacks had changed. The valued snack was always presented first. After entering the scanner again, participants watched a movie for 2 minutes with the devalued snack infested with waxworms. Next, the following instructions were shown: “You have found one of the original vending machines. As before you can tilt the machine for different snacks but you won’t be shown any snacks on screen. The amount earned will be recorded. Try to get as many snacks as you want as this will determine what you will eat afterwards!”

***Relapse rates***

After six months, AUDs were contacted about their alcohol use in the period since participation. We were able to reach 45 out of the total 51 AUDs (89%). AUDs were labeled as ‘relapsers’ if they reported two or more relapse days. Number of relapse days ranged from 2 to 95 days (mean=30.9 ± 33.5). Three AUDs in the abstinent group experienced a one-day lapse, all others did not report any lapses.

**ROI analyses**

Region of interest (ROI) analyses were performed using the same masks previously used in (Morris et al., 2015), which were based on previous literature. The ROI mask used for specific and general PIT effects included spheres around the bilateral amygdala, bilateral putamen, the ventral striatum and the medial OFC. The ROI mask used for outcome-devaluation included spheres around the medial OFC, medial PFC and the bilateral caudate. Masks are available online (<https://neurovault.org/collections/ETZNNLKP/>). Following Garbusow et al. (Garbusow et al., 2016), we additionally performed ROI analyses by extracting the mean parameter estimates from the individual contrast images for the right and the left NAcc (derived from the IBA SPM71 atlas incorporated in the WFU PickAtlas V3.0.5 (Maldjian, Laurienti, Kraft, & Burdette, 2003).

**Overview of fMRI contrasts**

| **PIT effect** |  | **First level contrast** | **Second level** |
| --- | --- | --- | --- |
| **sPIT** | Between-subject | sPIT > ITI | Cov #same - #diff responses |
| **gPIT** | Between-subject | CS+ > CS- | Cov #CS+ - #CS- responses |
| **sPIT** | Within-subject | sPIT pmod > ITI pmod |  |
| **gPIT** | Within-subject | CS + pmod > CS- |  |

**Supplementary Table 1.** PIT: Pavlovian-to-Instrumental Transfer; gPIT: general transfer; sPIT: specific transfer; ITI: Intertrial Interval (active baseline); CS+: Conditioned Stimulus (S3) associated with a snack (O3) but not paired with a specific action; CS-: Conditioned Stimulus (S4) that did not earn a snack food (“empty”) and was not paired with a specific action; pmod: parametric modulator, weighting each trial with the number of responses during that trial to test for correlations with signal change across trials; Cov: covariate using global PIT rates (subject-level), testing for correlations with the signal change across subjects.

| **MNI coordinates of Regions-of-interest** | | | |  | |
| --- | --- | --- | --- | --- | --- |
| **Test** | **Region** | **x** | **y** | | **z** |
| **PIT ROI** | Right amygdala | 20 | -6 | | -18 |
|  | Left amygdala | -22 | -8 | | -18 |
|  | Right putamen | 28 | -3 | | -5 |
|  | Left putamen | -27 | -15 | | -3 |
|  | Ventral striatum | 4 | 8 | | -2 |
|  | Medial OFC | -4 | 38 | | -20 |
| **Outcome Devaluation ROI** | Medial OFC | -4 | 38 | | -20 |
|  | Medial PFC | 3 | 55 | | -8 |
|  | Right caudate | 10 | 12 | | 7 |
|  | Left caudate | -11 | 6 | | 12 |

**Supplementary Table 2.** MNI coordinates of Regions-of-interest. Coordinates are exactly similar to previously used ROIs which were based on previous literature (Table 2 in Morris et al., 2015).

**Supplementary results**

**BMI and smoking status**

Because BMI and smoking status differed significantly between the groups, we ran additional analyses to assess the impact of these factors on the main effects. BMI did not significantly correlate with the outcome-devaluation, specific or general PIT effect. Furthermore, repeated-measures ANCOVAs for each of the main outcome measures with BMI and smoking status as covariates showed that between-group differences remained non-significant.

**Similar food and hunger ratings across groups before training**


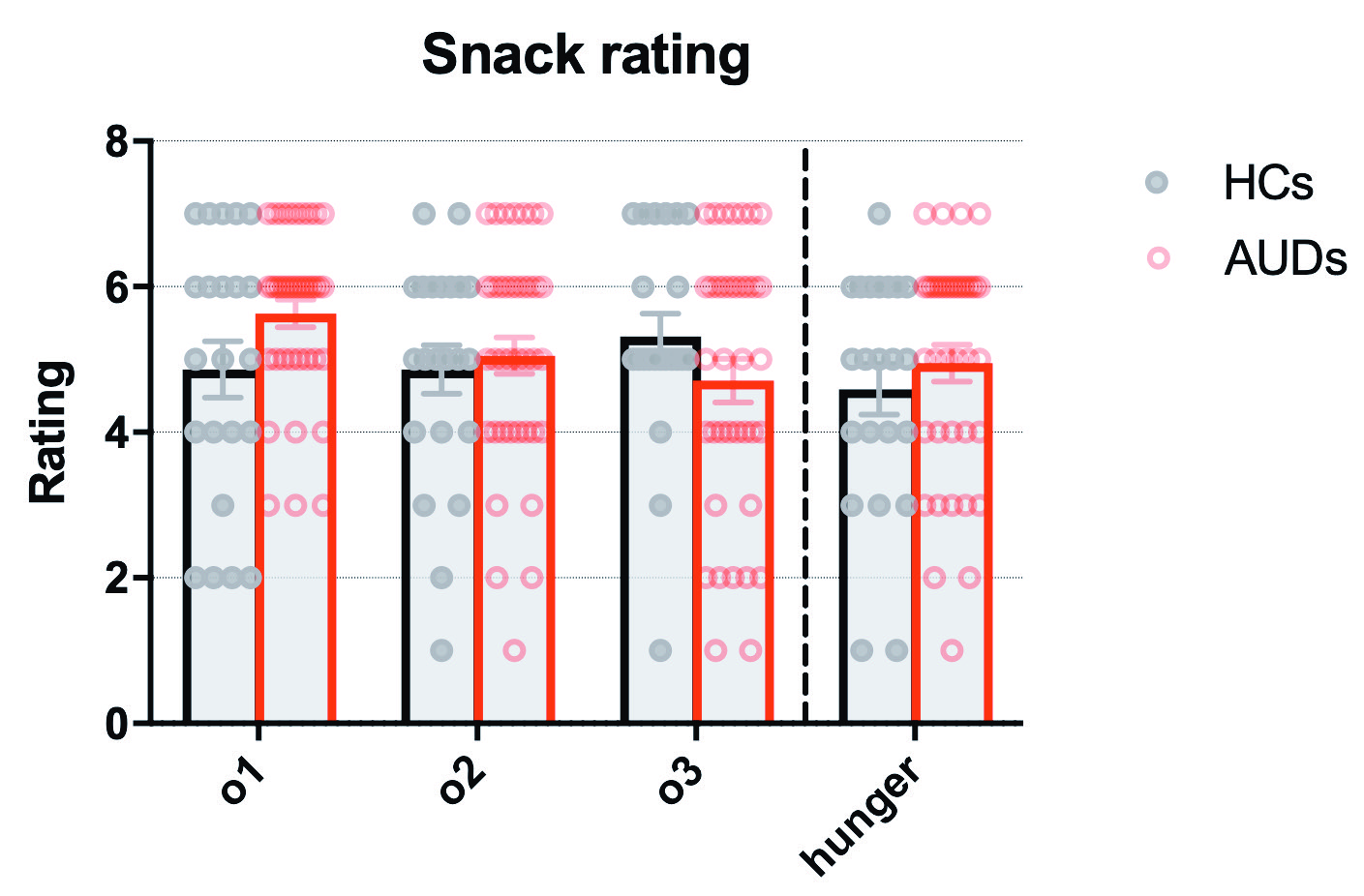


**Supplementary Figure 1**. Error bars represent SEM.

**Instrumental Conditioning**

|  | **AUD** | **HC** | **t(58)** | **p** |
| --- | --- | --- | --- | --- |
| **Total R1** | 126,4 (55,2) | 115,4 (38,0) | 0,83 | 0,41 |
| **Total R2** | 123,6 (58,1) | 100,0 (39,0) | 1,69 | 0,10 |
| **O1 won** | 9,9 (2,9) | 9,8 (2,8) | 0,07 | 0,95 |
| **O2 won** | 9,6 (3,5) | 8,2 (2,8) | 1,56 | 0,13 |

**Supplementary table 3.** Mean number (SD) of responses made and outcomes won during instrumental conditioning.

**Transfer test**

As expected, general PIT equally enhanced instrumental responses (no effect of response-type: p=0.12), nor did this differ between groups (response-type X group interaction: p=0.2).

**Rating change after devaluation**

Supplementary figure 2 shows the mean rating change (post rating - pre rating) for the valued and devalued snack after the experiment. The outcome devaluation procedure was successful, as revealed by a main effect of devaluation on rating change in both groups (F_1,53_=75.40; p<0.001). There was also a significant main effect of group (F_1,53_=6.73; p=0.01), driven by overall higher post-pre ratings in HCs. No significant group by devaluation interaction (F_1,53_=1.51; p=0.22) was observed. Importantly, devaluation significantly changed the rating of the devalued snack in both AUDs (p<0.001) and HCs (p=0.002). Pre- and post-ratings were similar for the valued snack in AUDs (p=0.25), but became significantly higher in HCs (p=0.03).

A two-sample t-test revealed that hunger ratings were similar between groups after the experiment (F_(2,57)_=0.35; p=0.70).


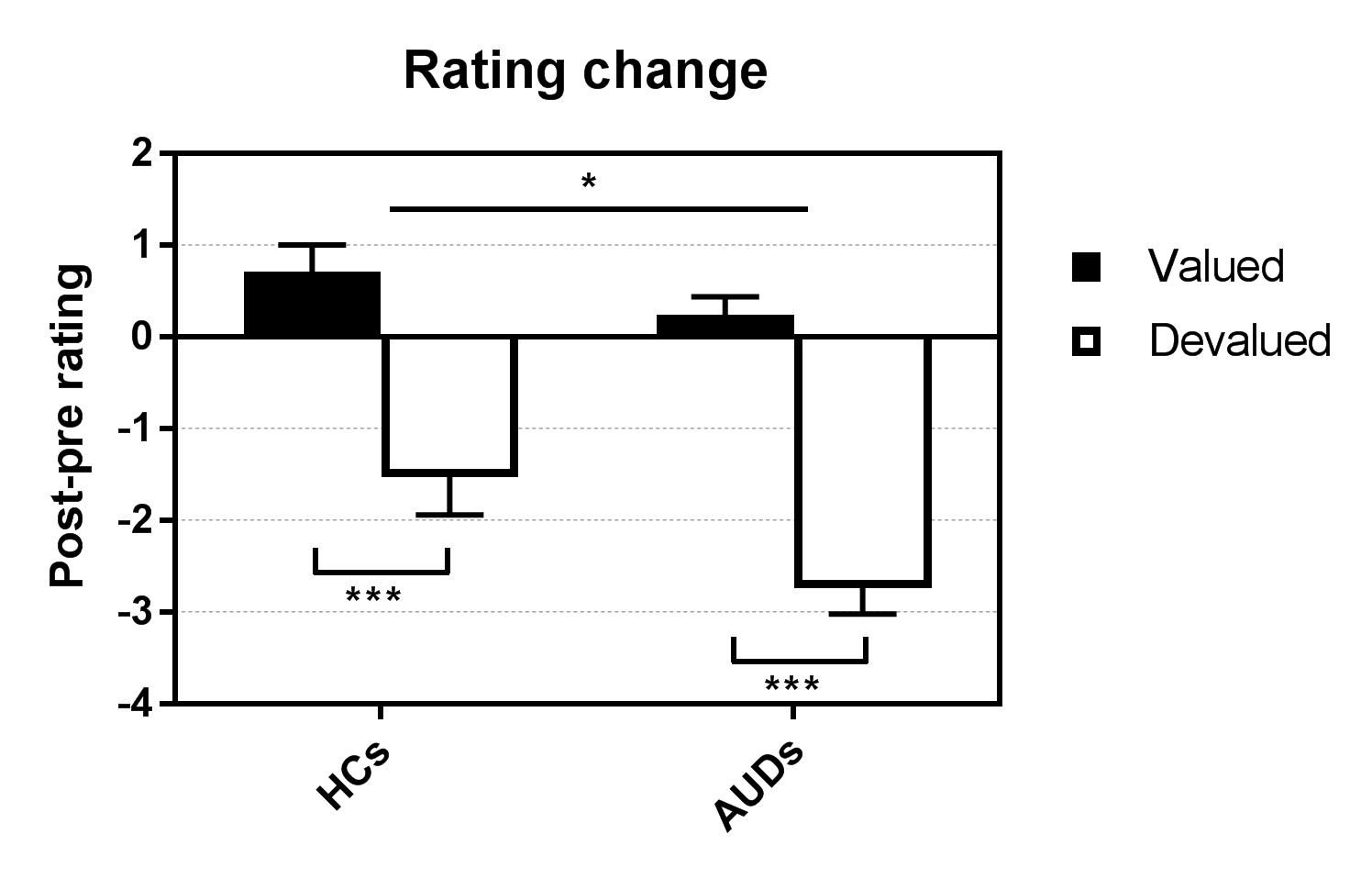


**Supplementary Figure 2.** *p<0.05; ***p<0.001. Error bars represent SEM.


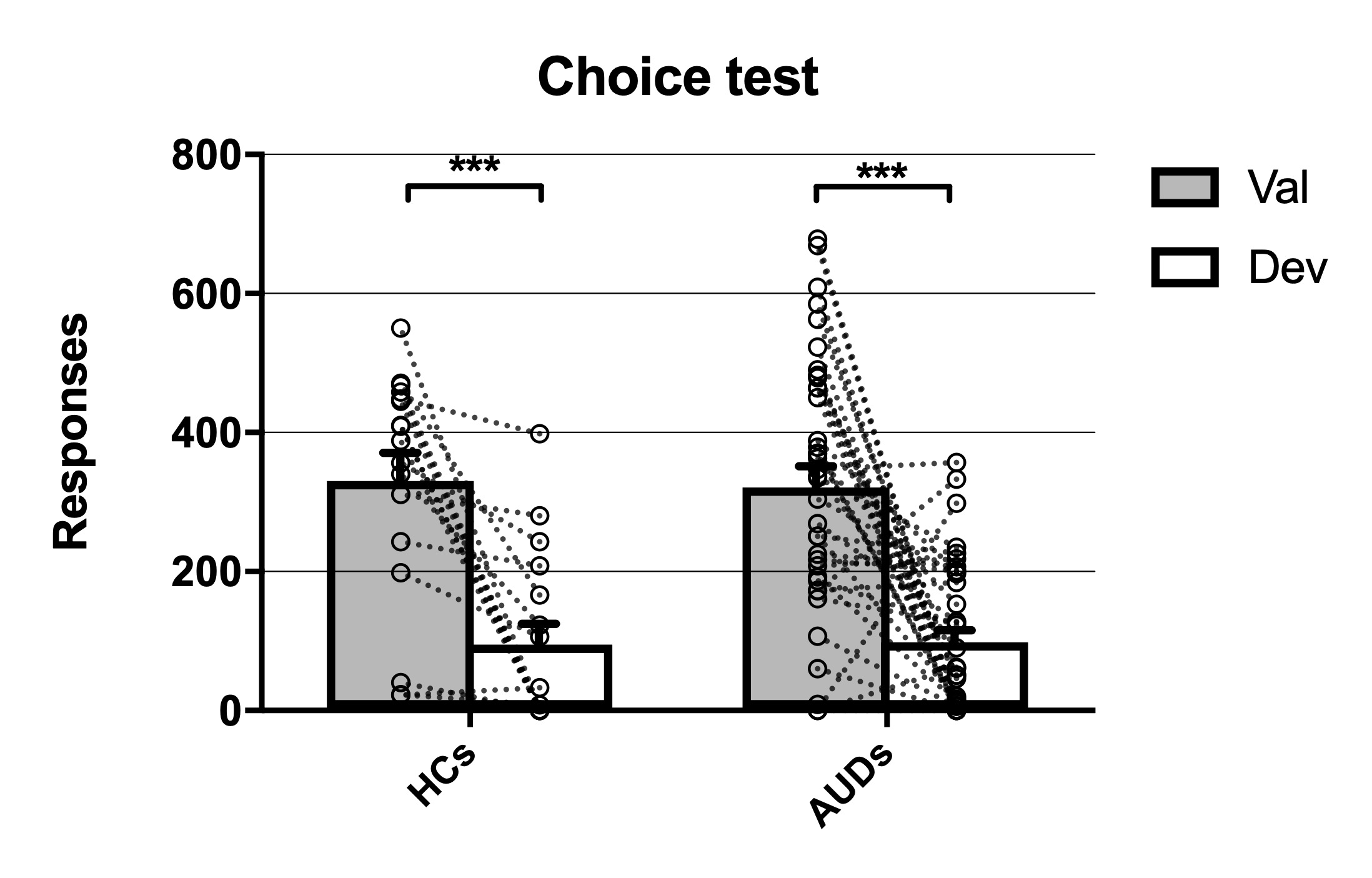


**Supplementary Figure 3**. Number of valued and devalued responses on the choice task following the devaluation procedure. Error bars represent SEM. The number of responses in AUDs (range valued=0–703, range devalued=0–365) and HCs (range valued=22–568, range devalued=0–410) for each condition did not differ between (F1,53=0.00; p<0.99) or interact with group (F1,53=0.00; p<0.92).


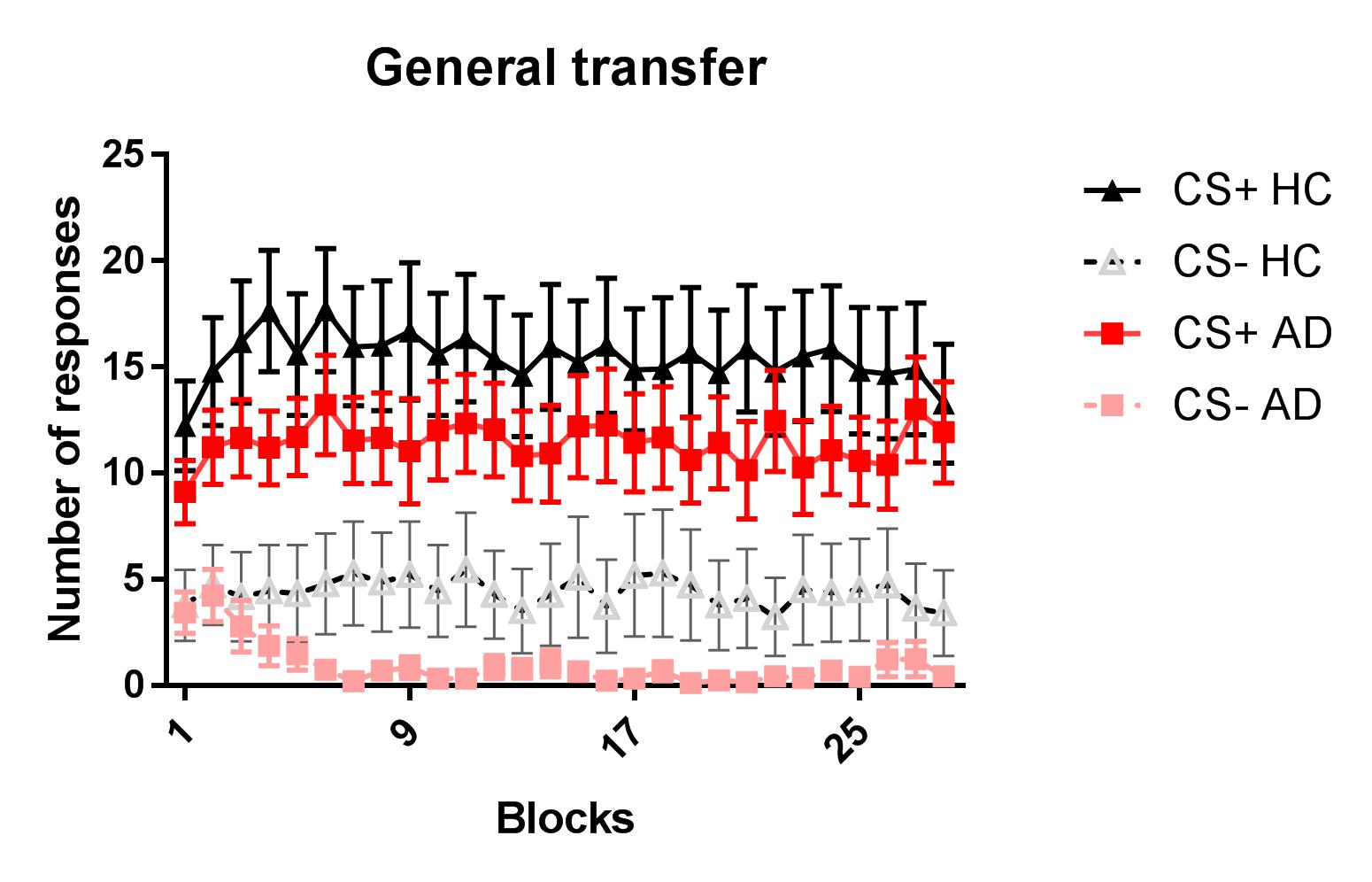


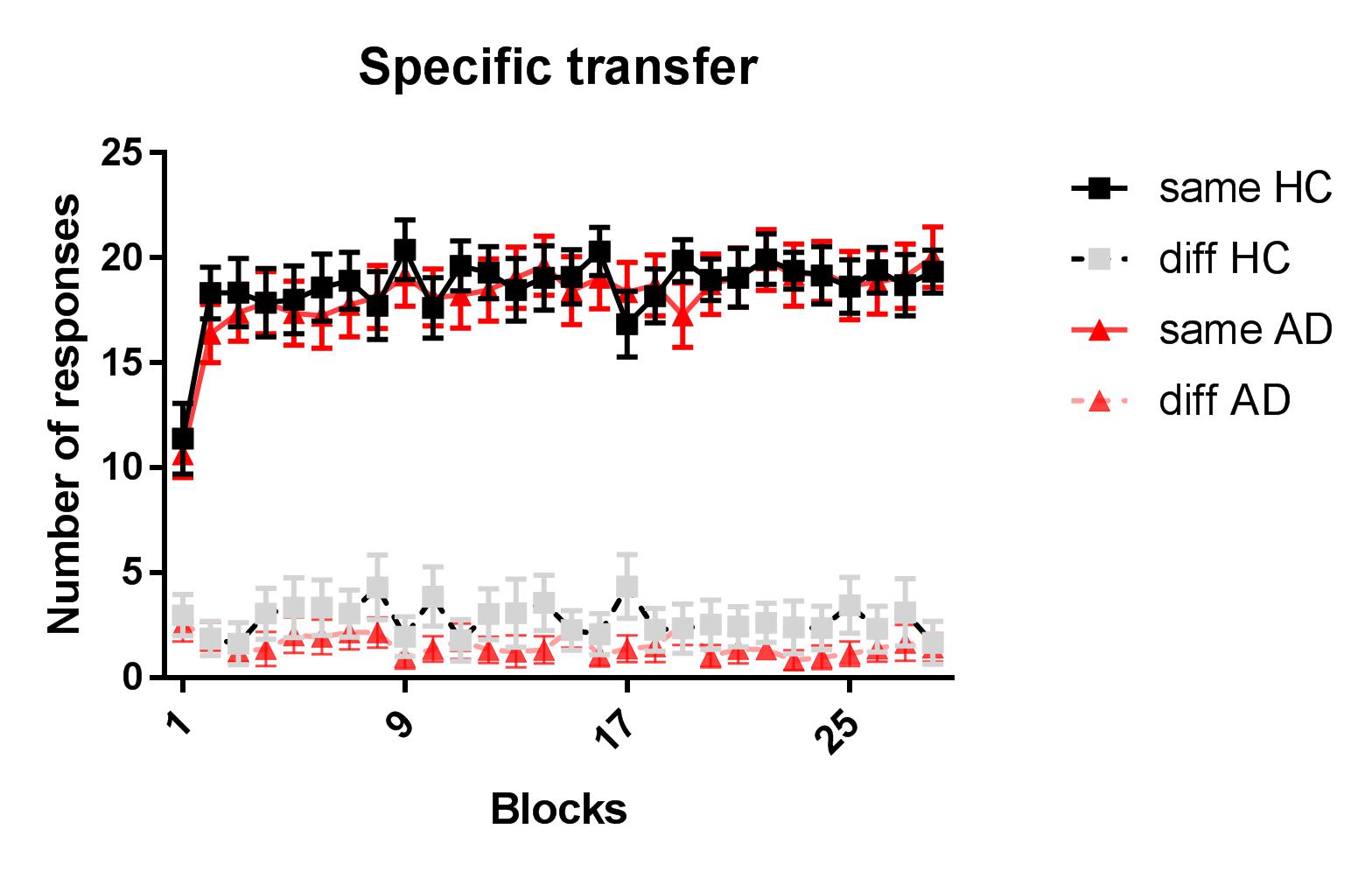


**Supplementary Figure 4**. Development of responding across all trials. Mean number of responses made for each 6-second trial (‘block’) during general and specific transfer.


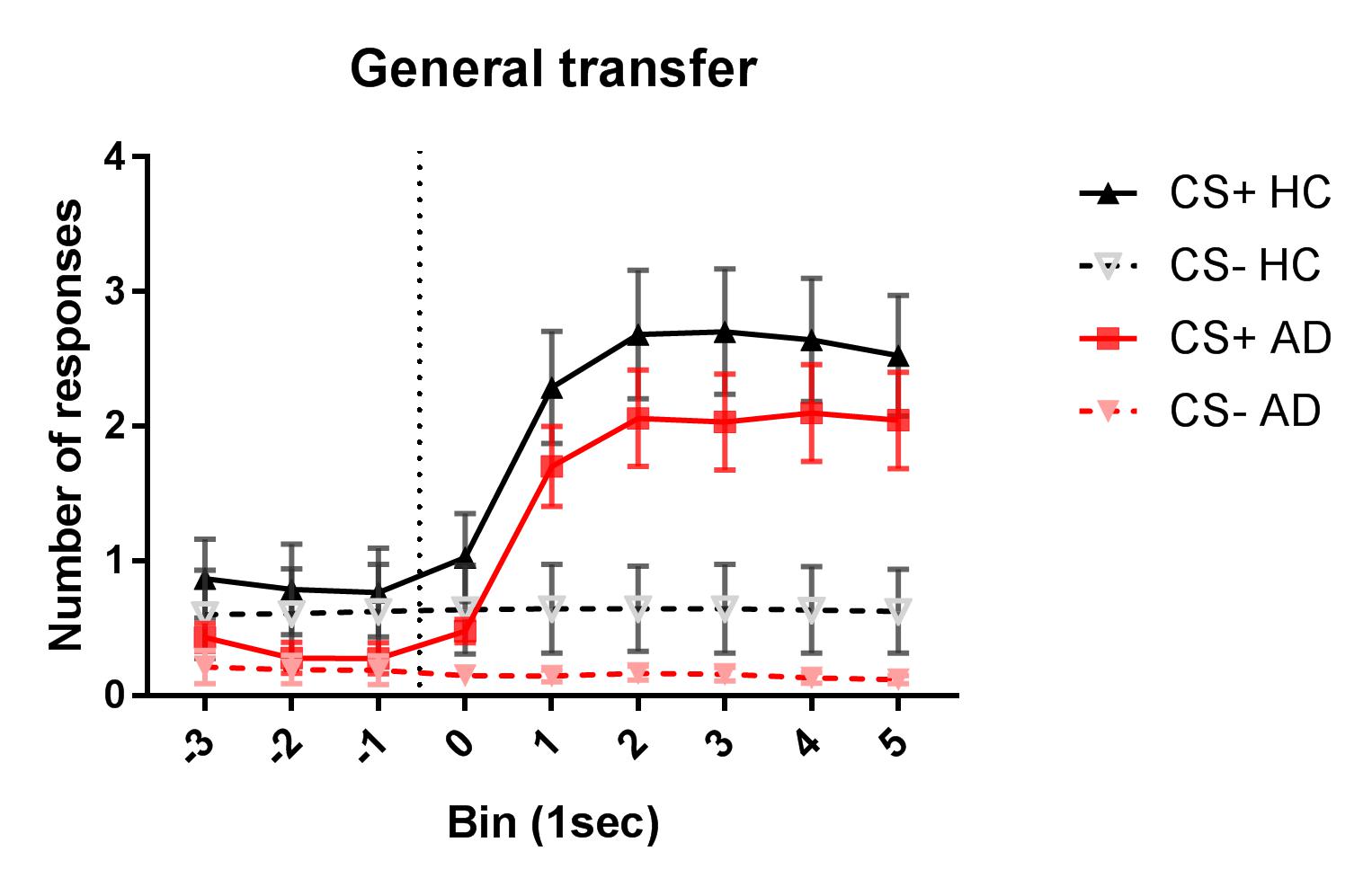


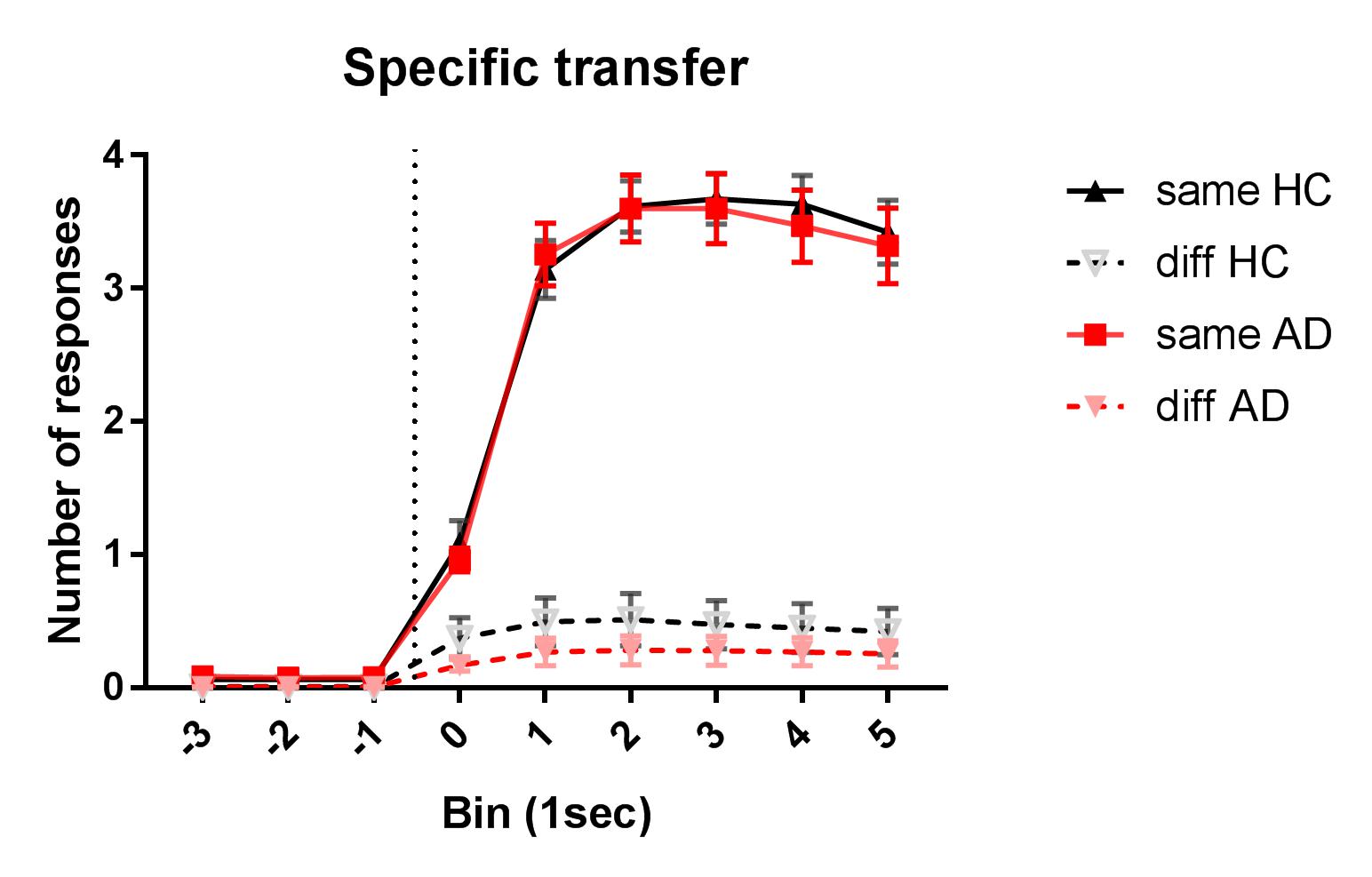


**Supplementary Figure 5**. Development of responding within trials. Average number of responses made within trials, plotted over time in 1 second bins for general and specific transfer.


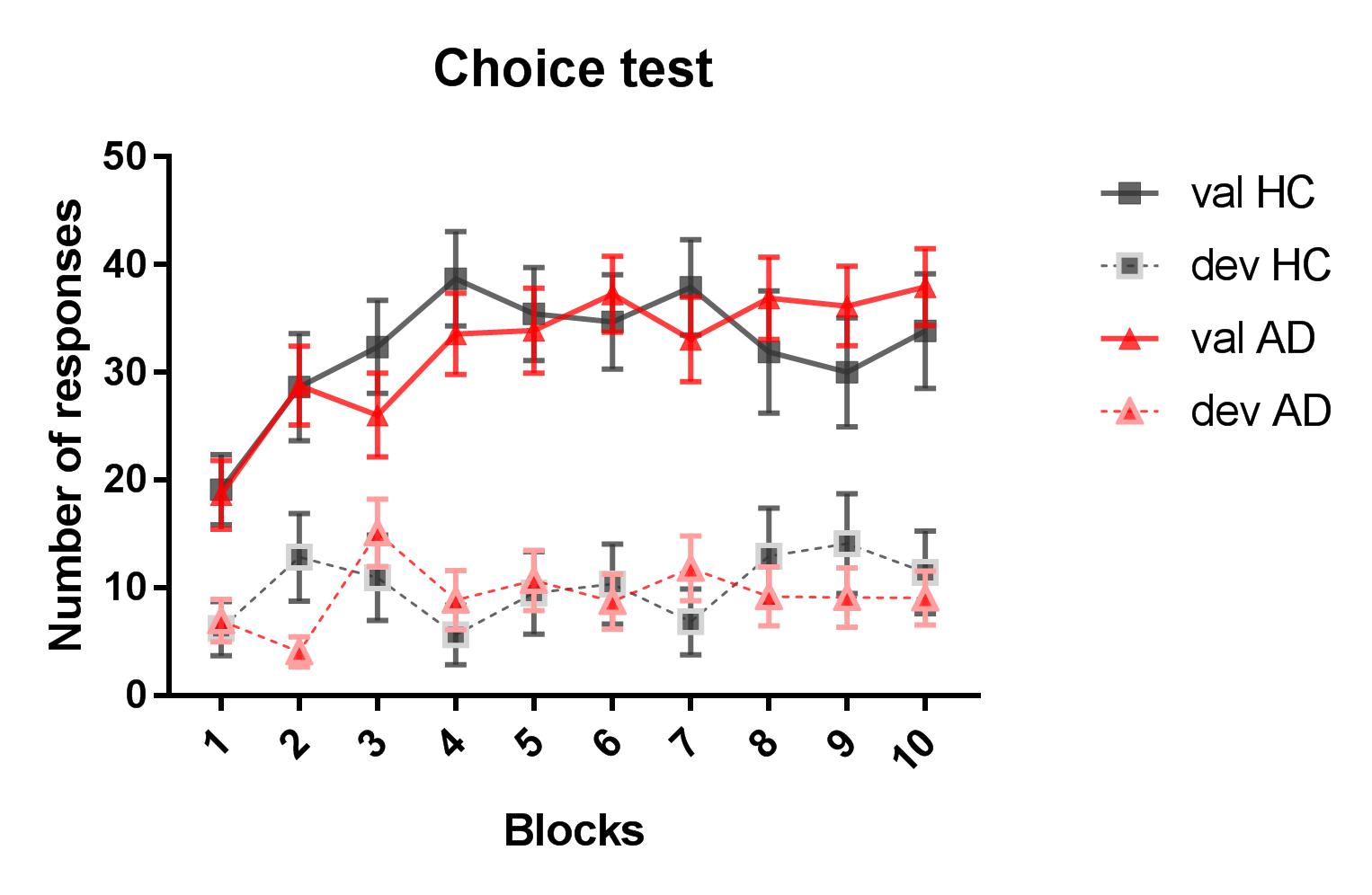


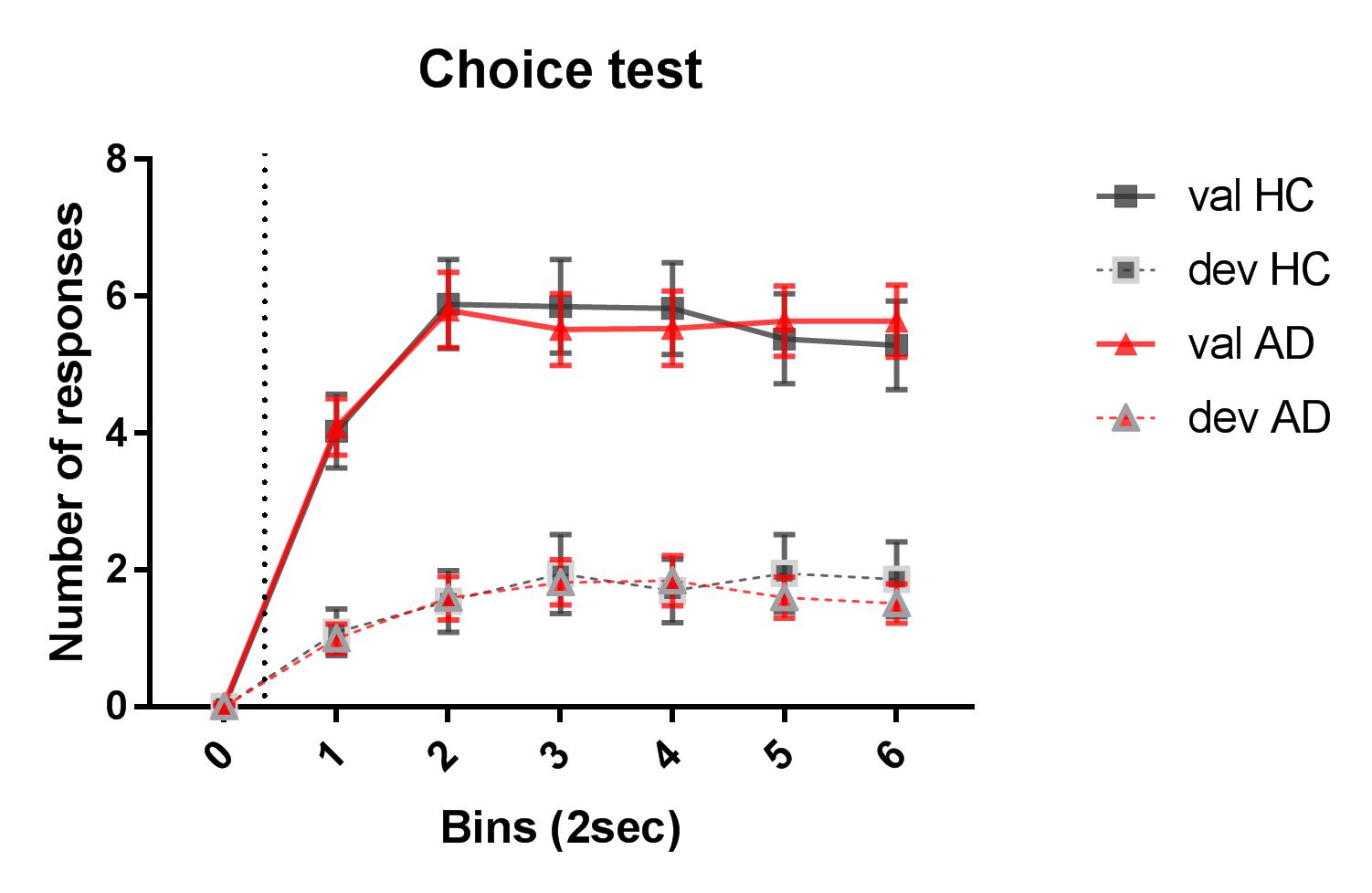


**Supplementary Figure 6**. Development of responding between and within blocks during the choice test following outcome devaluation.

**
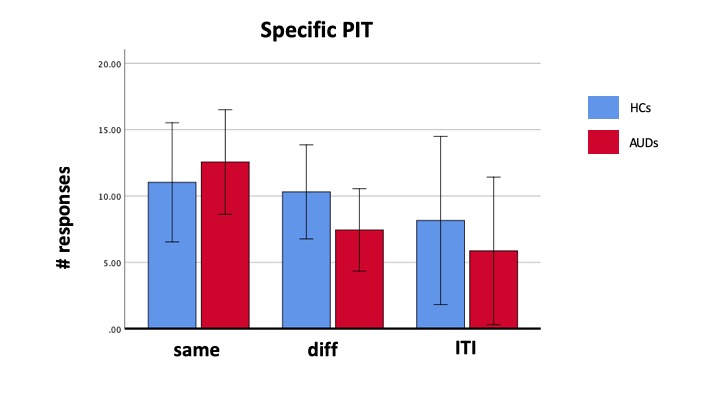
**

**Supplementary Figure 7**. Specific PIT in only those subjects (13 individuals with AUD and 10 HCs) who did not retain all contingencies correctly There was no significant specific PIT effect (p=0.09), nor did this differ between groups (p=0.19). Error bars represent 95% CI.


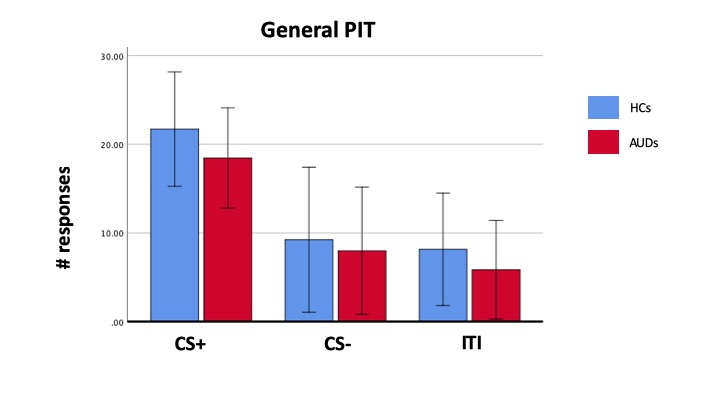


**Supplementary Figure 8**. Subjects (13 individuals with AUD and 10 HCs) who did not retain all contingencies correctly did show a significant General PIT effect (p<0.001), but this did not differ between groups (p=0.94). Error bars represent 95% CI.


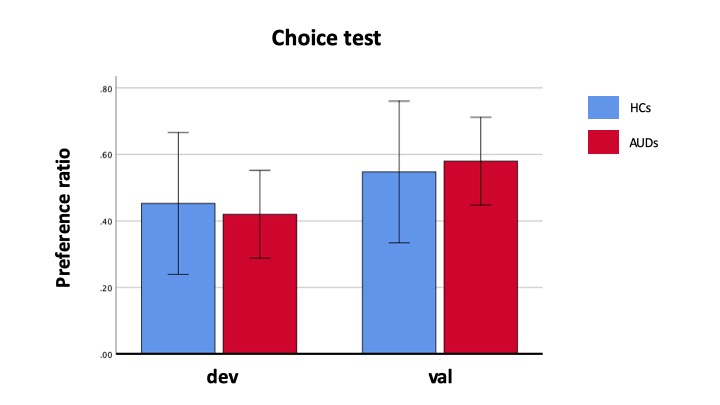


**Supplementary Figure 9**. Subjects who did not retain all contingencies correctly (13 individuals with AUD and 10 HCs) did not show a significant devaluation effect (p=0.30), nor did this differ between groups (p=0.79). Error bars represent 95% CI.

**Bayesian Repeated Measures ANOVAs**

## * Note that in the manuscript we report the BF _01_ values, which are computed from the BF_10_ and BF _Inclusion_ values reported in the tables: BF _01_ = 1 / BF_10_.

**Specific PIT effect**

| **Model Comparison** | | | | | | | | | | |  |
| --- | --- | --- | --- | --- | --- | --- | --- | --- | --- | --- | --- |
| **Models** | | **P(M)** | | **P(M\|data)** | **BF _M_** | | **BF _10_** | | **error %** | |  |
| Null model (incl. subject) |  | 0.200 |  | 6.539e -33 |  | 2.615e -32 |  | 1.000 |  |  |  |
| Response type (same vs diff) |  | 0.200 |  | 0.604 |  | 6.092 |  | 9.232e +31 |  | 0.681 |  |
| group |  | 0.200 |  | 2.013e -33 |  | 8.052e -33 |  | 0.308 |  | 2.167 |  |
| Response type + group |  | 0.200 |  | 0.342 |  | 2.075 |  | 5.224e +31 |  | 7.473 |  |
| Response type + group + Response type✻group |  | 0.200 |  | 0.055 |  | 0.232 |  | 8.376e +30 |  | 1.803 |  |
|  | | | | | | | | | | |  |

| **Analysis of Effects** | | | | | | | |
| --- | --- | --- | --- | --- | --- | --- | --- |
| **Effects** | | **P(incl)** | | **P(incl\|data)** | | **BF _Inclusion_** | |
| RM Factor 1 |  | 0.400 |  | 0.945 |  | 1.105e +32 |  |
| group_2 |  | 0.400 |  | 0.342 |  | 0.566 |  |
| RM Factor 1  ✻  group_2 |  | 0.200 |  | 0.055 |  | 0.160 |  |
|  | | | | | | | |

**General PIT effect**

| **Model Comparison** | | | | | | | | | | | |  |
| --- | --- | --- | --- | --- | --- | --- | --- | --- | --- | --- | --- | --- |
| **Models** | | **P(M)** | | **P(M\|data)** | **BF _M_** | | **BF _10_** | | **error %** | | |  |
| Null model (incl. subject) |  | 0.200 |  | 3.354e -12 |  | 1.342e -11 |  | 1.000 |  |  |  | |
| Response type (CS+ vs CS-) |  | 0.200 |  | 0.433 |  | 3.049 |  | 1.290e +11 |  | 1.023 |  | |
| group |  | 0.200 |  | 3.235e -12 |  | 1.294e -11 |  | 0.964 |  | 0.702 |  | |
| Response type + group |  | 0.200 |  | 0.514 |  | 4.238 |  | 1.534e +11 |  | 1.858 |  | |
| Response type + group + Response type✻group |  | 0.200 |  | 0.053 |  | 0.224 |  | 1.580e +10 |  | 1.453 |  | |
|  | | | | | | | | | | | |  |

| **Analysis of Effects** | | | | | | | |
| --- | --- | --- | --- | --- | --- | --- | --- |
| **Effects** | | **P(incl)** | | **P(incl\|data)** | | **BF _Inclusion_** | |
| RM Factor 1 |  | 0.400 |  | 0.947 |  | 1.437e +11 |  |
| group_2 |  | 0.400 |  | 0.514 |  | 1.189 |  |
| RM Factor 1  ✻  group_2 |  | 0.200 |  | 0.053 |  | 0.103 |  |
|  | | | | | | | |

| **Outcome devaluation**  **Model Comparison** | | | | | | | | | | | |  |
| --- | --- | --- | --- | --- | --- | --- | --- | --- | --- | --- | --- | --- |
| **Models** | | **P(M)** | | **P(M\|data)** | **BF _M_** | | **BF _10_** | | **error %** | | |  |
| Null model (incl. subject) |  | 0.200 |  | 8.175e -13 |  | 3.270e -12 |  | 1.000 |  |  |  | |
| Response type (CS+ vs CS-) |  | 0.200 |  | 0.753 |  | 12.186 |  | 9.209e +11 |  | 1.026 |  | |
| group |  | 0.200 |  | 1.931e -13 |  | 7.726e -13 |  | 0.236 |  | 0.956 |  | |
| Response type + group |  | 0.200 |  | 0.191 |  | 0.946 |  | 2.340e +11 |  | 3.404 |  | |
| Response type + group + Response type✻group |  | 0.200 |  | 0.056 |  | 0.236 |  | 6.827e +10 |  | 4.127 |  | |
|  | | | | | | | | | | | |  |

| **Analysis of Effects** | | | | | | | |
| --- | --- | --- | --- | --- | --- | --- | --- |
| **Effects** | | **P(incl)** | | **P(incl\|data)** | | **BF _Inclusion_** | |
| RM Factor 1 |  | 0.400 |  | 0.944 |  | 9.342e +11 |  |
| group_2 |  | 0.400 |  | 0.191 |  | 0.254 |  |
| RM Factor 1  ✻  group_2 |  | 0.200 |  | 0.056 |  | 0.292 |  |
|  | | | | | | | |

**fMRI results**

**Con2: Specific PIT between subjects, within ROI. Peak-level FWE corrected**

| **Region** | **L/R** | **X** | **Y** | **Z** | **k-extent** | **p(FWE-corr)** | **T** | **Z** |
| --- | --- | --- | --- | --- | --- | --- | --- | --- |
| **Caudate** | R | 9 | 11 | -1 | 31 | 0.009 | 5.46 | 4.86 |
| **Putamen*** | R | 33 | -7 | -4 | 70 | 0.009 | 5.46 | 4.86 |
| **Putamen*** | R | 27 | 2 | -1 |  | 0.027 | 5.10 | 4.59 |

* extends to right pallidum

**Con3: General PIT between subjects, within ROI. Peak-level FWE corrected**

| **Region** | **L/R** | **X** | **Y** | **Z** | **k-extent** | **p(FWE-corr)** | **T** | **Z** |
| --- | --- | --- | --- | --- | --- | --- | --- | --- |
| **Putamen** | R | 33 | -1 | -1 | 79 | 0.000 | 7.41 | 6.14 |
| **Pallidum** | R | 24 | -1 | -1 | 21 | 0.000 | 7.32 | 6.09 |
| **Putamen** | L/R | -30 | -10 | -1 | 56 | 0.047 | 4.91 | 4.45 |

**Con6: General PIT, within subject. Whole brain FWE corrected**

| **Region** | **L/R** | **X** | **Y** | **Z** | **k-extent** | **p(FWE-corr)** | **T** | **Z** |
| --- | --- | --- | --- | --- | --- | --- | --- | --- |
| **mOFC** | R | 12 | 38 | -10 | 28 | 0.001 | 6.16 | 5.32 |
|  | L | -12 | 38 | -10 | 22 | 0.005 | 5.71 | 5.01 |
| **ACC** | R | 12 | 35 | 2 | 2 | 0.028 | 5.17 | 4.62 |

mOFC=medial OrbitoFrontal Cortex; ACC=Anterior Cingulate Cortex

**Con9: General PIT, within subject. Peak-level FWE corrected**

| **Region** | **L/R** | **X** | **Y** | **Z** | **k-extent** | **p(FWE-corr)** | **T** | **Z** |
| --- | --- | --- | --- | --- | --- | --- | --- | --- |
| **Thalamus** | R&L | 3 | -10 | 2 | 88 | 0.036 | 5.1 | 4.57 |
| **SMA** | L | -9 | 11 | 50 | 128 | 0.017* | 4.55 | 4.15 |
| **Insula** | R | 33 | 23 | 2 | 95 | 0.046* | 4.17 | 3.86 |

## SMA=Suppl Motor Area; *=FWE cluster-corrected

## Supplementary references

Conigrave, K. M., Hall, W. D., & Saunders, J. B. (1995). The AUDIT questionnaire: choosing a cut-off score. Alcohol Use Disorder Identification Test. *Addiction (Abingdon, England)*, *90*(10), 1349–1356. Retrieved from http://www.ncbi.nlm.nih.gov/pubmed/8616463

Garbusow, M., Schad, D. J., Sebold, M., Friedel, E., Bernhardt, N., Koch, S. P., … Heinz, A. (2016). Pavlovian-to-instrumental transfer effects in the nucleus accumbens relate to relapse in alcohol dependence. *Addiction Biology*, *21*(3), 719–731. https://doi.org/10.1111/adb.12243

Maldjian, J. A., Laurienti, P. J., Kraft, R. A., & Burdette, J. H. (2003). An automated method for neuroanatomic and cytoarchitectonic atlas-based interrogation of fMRI data sets. *NeuroImage*, *19*(3), 1233–1239. https://doi.org/10.1016/S1053-8119(03)00169-1

Morris, R. W., Quail, S. L., Griffiths, K. R., Green, M. J., & Balleine, B. W. (2015). Corticostriatal Control of Goal-Directed Action Is Impaired in Schizophrenia. *Biological Psychiatry*, *77*(2), 187–195. https://doi.org/10.1016/j.biopsych.2014.06.005

Peirce, J. W. (2007). PsychoPy—Psychophysics software in Python. *Journal of Neuroscience Methods*, *162*(1–2), 8–13. https://doi.org/10.1016/j.jneumeth.2006.11.017

Schmand, B., Bakker, D., Saan, R., & Louman, J. (1991). [The Dutch Reading Test for Adults: a measure of premorbid intelligence level]. *Tijdschrift Voor Gerontologie En Geriatrie*, *22*(1), 15–19. Retrieved from http://www.ncbi.nlm.nih.gov/pubmed/1877068

Wechsler, D. (1981). WAIS-R Manual. *The Psychological Corp, San Antonio*.
